# Pathways for socio-economic system transitions expressed as a Markov chain

**DOI:** 10.1371/journal.pone.0288928

**Published:** 2023-07-31

**Authors:** Vanessa Jine Schweizer, Alastair David Jamieson-Lane, Hua Cai, Stephan Lehner, Matteo Smerlak

**Affiliations:** 1 Climate and Global Dynamics Division, National Center for Atmospheric Research, Boulder, Colorado, United States of America; 2 Department of Knowledge Integration, Faculty of Environment, University of Waterloo, Waterloo, Ontario, Canada; 3 Mathematics, University of British Columbia, Vancouver, British Columbia, Canada; 4 Institute for Chemistry and Biology of the Marine Environment, Campus Wechloy, University of Oldenburg, Oldenburg, Lower Saxony, Germany; 5 School for Environment and Sustainability, University of Michigan, Ann Arbor, Michigan, United States of America; 6 Department of Civil and Environmental Engineering, University of Michigan, Ann Arbor, Michigan, United States of America; 7 Institute of Air Transportation Systems, German Aerospace Center (DLR), Hamburg, Germany; 8 Perimeter Institute for Theoretical Physics, Waterloo, Ontario, Canada; Robert Koch-Institut, ISLAMIC REPUBLIC OF IRAN

## Abstract

Cross-impact balance (CIB) analysis provides a system-theoretical view of scenarios useful for investigating complex socio-economic systems. CIB can synthesize a variety of qualitative or quantitative inputs and return information suggestive of system evolution. Current software tools for CIB are limited to identifying system attractors as well as describing system evolution from only one scenario of initial conditions at a time. Through this study, we enhance CIB by developing and applying a method that considers all possible system evolutions as transitions in a Markov chain. We investigated a simple three-variable system (27 possible scenarios) of the demographic transition and were able to generally replicate the findings of traditional CIB. Through our experiments with four possible approaches to produce CIB Markov chains, we found that information about transition pathways is gained; however, information about system attractors may be lost. Through a comparison of model results to a recent literature review on human demography, we found that low-income countries are more likely to remain stuck in a demographic trap if economic development is not prioritized alongside educational gains. Future work could test our comparative methodological findings for systems comprised of more than three variables.

## 1. Introduction

Scenarios are a central fixture of futures studies in a variety of contexts ranging from business planning (e.g., [1]), to risk analysis [2], to sustainability studies [3]. Some scenario exercises include detailed quantitative analysis through systems dynamics models (e.g., [4]), economic models (e.g., [5]), or agent-based models [6]. However, before employing such powerful quantitative tools, analysts and decision-makers often narrow the number of cases quantitative models will investigate to a handful of instances. If not done thoughtfully, this selection process can have the undesirable effect of artificially constraining the types of futures considered [7], which, at worst, can eliminate futures from investigation altogether that are relevant for decision-making. Systematic approaches to exploring large spaces of scenarios have been developed, and in this project, we focus on modifying one method in particular—cross-impact balance analysis, or CIB [8]. CIB analysis has been applied to over 100 studies in dozens of contexts [9] including innovation studies [10], strategies for public health [11], resource management [12], and the human dimensions of climate change [7, 13].

This study focuses on CIB because it is the one approach that employs a system theoretical view to the *evolution* of scenarios for the purpose of identifying a small number of interesting cases (to achieve the same end, other methods rely on subjective probability judgments [14, 15] or statistical data mining [16]). In CIB, a system theoretical approach is used to generate measures of internal consistency for individual scenarios, where such scenarios embody a self-consistent set of relationships among scenario factors. The ability of CIB to provide a measure for internal consistency has been recognized as an important methodological advancement for futures studies [17]. Building on this particular feature, we see additional potential for the analytical power of CIB. This is because information that is used to find internally consistent scenarios can also be used to trace the evolution of scenarios with weaker internal consistency to progressively more consistent scenarios. Through this study, we developed extensions to traditional CIB analysis to consider four research objectives:

- The development of an approach for the simultaneous visualization of multiple scenarios with high internal consistency, as well as the pathways of less consistent scenarios that transition to them;
- The development of update rules for inconsistent scenarios that are stochastic, complementing traditional CIB update rules, which are deterministic;
- A comparison of the findings for system behaviors across various update rules to examine any sensitivities in the results;
- The application of standard measures from Information Theory to qualitatively describe the results of different stochastic update rules. For example, Shannon entropy [18] can be used to meaningfully quantify notions such as “uncertainty” and “unpredictability”.

We address these questions by first reviewing the original CIB analysis of Weimer-Jehle [8] in section 2. In particular, we note that the evolutionary dynamics of CIB can be formulated as a Markov chain. This observation then leads us in section 3 to put forward a stochastic generalization of the CIB algorithm, yielding probabilistic rather than absolute forecasts. In section 4, we use traditional and modified CIB algorithms to analyze a test case of a simple system for the demographic transition comprised of three variables. In section 5, we discuss similarities and differences in our findings based on traditional (deterministic) CIB update rules and stochastic modifications. We also compare analytical findings to a recent review of the literature on human demography. A summary of our conclusions and comments on future work appear in section 6. We find that by visualizing a full set of scenarios and exploring their evolution comprehensively, we are able to gain new information about stable, internally consistent scenarios (i.e., system attractors) and the paths of unstable scenarios that transition to them.

## 2. Material and methods: Cross-impact balances (CIB)

CIB analysis was developed to improve existing cross-impact tools [14, 15] for examining the combined effects of multiple factors that could affect the behavior of a system, such as cultural, environmental, economic, or political influences in social systems. In this section, we provide an overview of the CIB method, its implementation, typical rules for updating scenarios with weak internal consistency, and limitations. We also introduce the concept of representing system evolution (i.e. updates according to a set of rules) in CIB as a Markov chain. For more comprehensive studies of the CIB method, please refer to Weimer-Jehle [8, 19–21].

### 2.1. The cross-impact matrix in CIB

The constituent factors of scenarios in CIB are called *descriptors*. A descriptor is some aspect of the system or world being described in the scenario. Throughout this paper, we refer to a three-descriptor example discussed by Schweizer and O’Neill [13]. The example system is a simple representation of a social system, where the descriptors are conceptualized at a global or large continental-region level: population, income per capita (measured as GDP per capita) and educational attainment.

Descriptors can be in one of any number of *states*. In the above example, these could be “High,” “Medium,” or “Low” to correspond with alternative outcomes for long-term trends. Any system of interest will include some number of descriptors, each with their own set of states. Descriptors need not all have the same number of states. In CIB analysis, each unique combination of states (one state per descriptor) is called a “scenario”. For our example system, a scenario would be: low population, high income per capita, high educational attainment.

Once the relevant descriptors and states have been identified, we must next estimate the relationships between states of different descriptors. For example, does the educational state “High” have an influence on the population state “Low”? If so, what is the nature of this influence, and how strong is it? Assessing such influences requires making judgments about whether a particular descriptor state promotes, restricts or has no effect on some other possible state of the system. This effect can be recorded as an integer in a matrix. This matrix is referred to as a *cross-impact matrix*, and the quantified influence of one state on another is a *cross-impact judgment*. In CIB, positive integers indicate that a descriptor state is promoted; negative integers indicate a descriptor state is restricted; a cross-impact judgment of 0 indicates no effect. The interval of such judgments is typically [–3,3]; however, a matrix that has undergone standardization [22] may contain integers with absolute values > 3. Cross-impact matrices are not unique to CIB. They are also used for cross-impact analysis [14] and morphological analysis [15]. Nevertheless differences across these methods result in different uses of the values recorded in a cross-impact matrix as well as differences in analytical capabilities.

**Fig 1:**
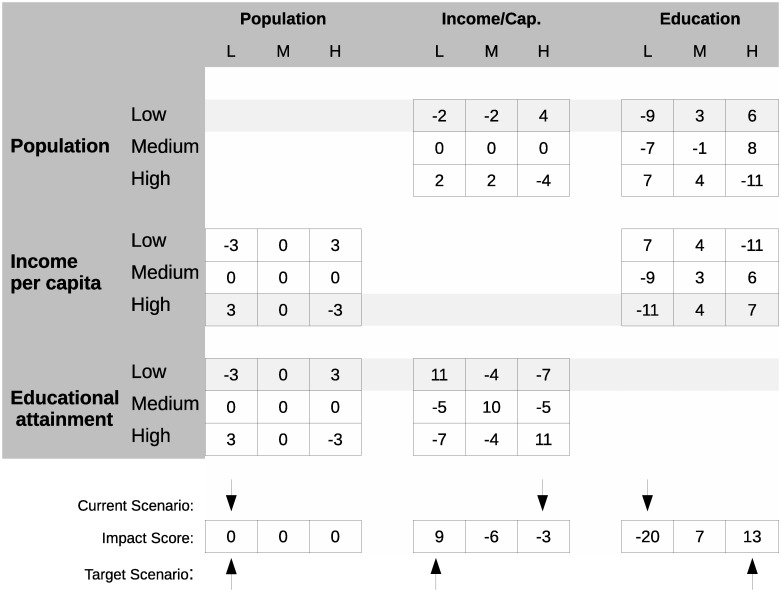
A typical CIB matrix standardized with ScenarioWizard [22], with three descriptors and three states per descriptor. Descriptors are for a social system conceptualized at a global or large continental-region level, i.e. an aggregation of countries. The top middle submatrix gives the (estimated) directed influences from descriptor “1” (Population) upon descriptor “2” (Income per capita) with the top right entry of this submatrix (“4”), denoting the extent to which Low population promotes high income. Rows highlighted in grey represent the possible scenario “Low population, High income per capita, Low educational attainment”. Below the impact matrix we sum these rows and determine which “target” scenario is most supported by the current scenario. Figure adapted with permission from Lloyd and Schweizer [23].

The CIB matrix of the 3-descriptor Population-Income-Education case study is presented in Fig 1. The rows of this matrix indicate that a given state of a descriptor causes a particular influence on each column of the grid, where columns represent the descriptor states being influenced (i.e., receiving influences), sometimes referred to as “target states” [13]. These influences between different descriptors (cross-impact judgments) can be gathered by eliciting judgments from experts in relevant fields [13], literature review [7], or even quantitative data such as correlation coefficients. The numerical values of the CIB matrix used in this study were derived from an expert elicitation involving human participants [13], which makes this case study akin to a retrospective study of archived samples. All data were fully anonymized before they were accessed. A detailed discussion of the meaning of the different descriptors and their states can be found in Section 3 in S1 File.

### 2.2. Internally consistent scenarios

In CIB analysis, the key property of a scenario is its internal *consistency*. To estimate it, *impact scores* of each descriptor state with respect to a given scenario are calculated. These are defined as follows.

Let z = [z_1_,z_2_,…] denote a scenario, such that for each descriptor *i*, the state is z_i_. Denoting C_i,j_(s,s’) the cross-impact of two states *s* and *s*′, the *impact score* of the state *s* of descriptor *i* with respect to z is the sum

$$\Theta_{i}\left(s;z\right)=\Sigma_{j}C_{j,i}\left(z_{j},s\right).$$ (1)

States with high impact scores are compatible with the given scenario; alternatively, states with low impact scores are less compatible, and states with negative impact scores are incompatible.

The *(absolute) consistency* γ_i_ of the descriptor *i* is then defined as the difference between the impact score of the scenario state z_i_ and the highest impact score of all other states of that descriptor, i.e.


$$\gamma_{i}=\Theta_{i}\left(z_{i};z\right)-max_{s\in i}\;\Theta_{i}\left(s;z\right).$$ (2)

The lowest consistency *min*_*i*_ γ_i_ of all descriptors of the scenario z is called the *(absolute) consistency* Γ(z) of scenario z. If Γ(z)≥ 0, we say that scenario z is perfectly internally consistent.

For example, in Fig 1 we consider a given 3-descriptor scenario of Low population, High income per capita and Low educational attainment. To determine the net effect of the given three descriptor states on the single state “Low educational attainment,” we examine the columnar sum corresponding to “(3) Education” and the state “L”. The highlighted cross-impact judgments are -9, -11 and 0 for the appropriate column. This means that, as a target state, “Low education” has an impact score of -20 and is strongly discouraged by the initially assumed scenario. Medium education is seen to have an impact score of 7, while the state High education has an impact score of 13. Furthermore, we see in this example that the population descriptor is consistent because the population is currently low, and “Low population” has the highest impact score among possible population states. However, the descriptor “Income per capita” is inconsistent, because “Low income” has a higher impact score than the initially assumed state “High”.

Measures of consistency matter because consistent scenarios represent situations of the system that can be considered stable [21, 24], or self-reinforcing. Inconsistent scenarios, on the other hand, represent situations that lend themselves to system corrections, or change. Traditionally, CIB is primarily used to identify scenarios possessing high internal consistency. However, it could be argued that the world itself is full of dynamic, ever-changing systems with no clear “final state”. This suggests that it may be worthwhile to investigate the evolution of internally *inconsistent scenarios*, since they are suggestive of how a system might correct internal inconsistencies. To do this, we must consider how an inconsistent scenario is updated, or in CIB parlance, “succeeded”, by another. It should be noted that succession analysis makes sense only when descriptors are reversible. Examples of reversible descriptors would be patterns of economic growth, or political parties that come to power. For the simple Population-Income-Education example discussed in this study, all descriptors are reversible. However, descriptors in CIB analysis can also be irreversible, such as whether a breakthrough occurs for an experimental technology. If a breakthrough occurs in one scenario, yet a successor scenario indicates that the breakthrough does not occur, the results of the succession analysis must be interpreted with caution.

### 2.3. CIB succession: Rules for updating inconsistent scenarios

To trace system pathways to internally consistent scenarios, Weimer-Jehle [8] introduced the concept of *CIB succession*. This consists of a predefined rule for how inconsistent descriptors are updated until an internally consistent scenario—a system *attractor*—is reached. We call this updating rule a *succession rule*. In some cases the system may not converge upon an attractor and may instead converge to a *cycle* of inconsistent states (cyclic attractor).

We would like to stress that succession analysis in the classical sense is designed simply as a tool for identifying internally consistent scenarios, which can also give a rough indication of their importance (via their basin of attraction [25, 26] or “attractor weight”, as discussed in section 5.1, Table 1). CIB succession can be performed with alternative rules, and the choice for what rule to apply is considered to be of secondary importance, as succession is not expected to necessarily represent real system behavior. Nevertheless, it remains an intriguing prospect that CIB might move from focusing on the self-consistency of scenarios fixed in time to considering how scenarios change through time. Potentially this could be achieved through enhancements to succession analysis.

**Table 1:** Comparison of notable scenarios for the Population-Income-Education system according to five succession rules.

Attractor scenarios: (Pop, Inc, Educ)	LHH	MHH	HLM↔HML	HMM	HLL
Succession rule:					
Global					
Attractor type Attractor weight^a^ Total impact score^b^	Final 15 34	N/A N/A N/A	Cyclic 8 8↔1	Final 3 19	Final 1 33
Adiabatic					
Attractor type Attractor weight^a^	Final 17	N/A N/A	N/A N/A	Final 4	Final 6
Random descriptor					
Attractor type Eigenvector centrality^c^, *x*_*v*_	Stable^d^ *x*_*v*_ = 1	Unstable^e^ (*p* = 0.67)	N/A	Unstable^e^ (*p* = 0.6)	Stable^d^
Boltzmann local					
Attractor type Eigenvector centrality^c^ Entropy (β = 1)	Stable^d^ *x*_*v*_ = 1	N/A	N/A	Unstable^e^ (*p* = 0.33)	Stable^d^ *x*_*v*_ < 0.002
	Rapid decreases in uncertainty				
Logistic local					
Attractor type Eigenvector centrality^c^ Entropy (β = 1)	Stable^d^ *x*_*v*_ = 1	Unstable^e^ (*p* = 0.42)	N/A	Unstable^e^ (*p* = 0.33)	Stable^d^ *x*_*v*_ < 0.02
	Slow decrease in uncertainty				
Arctan local					
Attractor type Eigenvector centrality^c^ Entropy (β = 1)	Unstable^e^ (*p* = 0.15) *x*_*v*_ = 1	Unstable^e^ (*p* = 0.17) *x*_*v*_ < 0.25	N/A	Unstable^e^ (*p* = 0.17) *x*_*v*_ < 0.25	Unstable^e^ (*p* = 0.17) *x*_*v*_ < 0.25
	Very Slow decrease in uncertainty				

Global and adiabatic succession are deterministic; Boltzmann, logistic, and arctan are stochastic. Deterministic succession rules assign scenarios to non-overlapping basins of attraction (attractor weights). Stochastic succession rules assign transition probabilities to scenarios and enable measurements of entropy.

^a^ The number of scenarios confined to a basin of attraction.

^b^ The sum of impact scores across all self-consistent descriptor states; see [8].

^c^ Indicated by the size of the node in the Markov chain.

^d^ Probability of self-looping is 0.95 or greater.

^e^ Probability of self-looping is 0.94 or lower.

In the software package ScenarioWizard [27], succession analysis is available for one parent scenario and all of its successors; however, it is not possible to visualize these successions, let alone that of multiple parent scenarios simultaneously. In section 3, we introduce how this limitation can be addressed. Explicit examples of results from alternative succession analyses are in results and discussions sections 4 and 5. In ScenarioWizard, the default and most straightforward succession rule is *global succession*. Under this rule, given a particular scenario, the impact scores of all states for all descriptors are considered, and the states that are most encouraged by the initially assumed scenario are selected. An inconsistent scenario will result in a set of new states describing a different scenario, which we call the successor of the previous scenario. Starting with any scenario and applying this rule repeatedly, one will either eventually reach a consistent scenario (which is its own successor) or find that scenarios recur in a loop of inconsistent scenarios, which succeed one another in turn.

A second rule is *local succession*. Similar to global succession, it searches for states that have the highest impact score. The algorithm then compares the absolute consistency of each descriptor and updates only the state of the descriptor with the lowest absolute consistency (the most inconsistent descriptor). Thus, any scenario and its successor will differ at most in one descriptor. Compared to global succession, local succession analysis is less prone to loops (cyclic attractors), although loops are still possible.

### 2.4. Visualizing CIB succession as a Markov chain

A Markov chain is a mathematical model of a dynamic system, where the probabilities of future system states are fully determined by the present system state (as opposed to all past states). For a full introduction to Markov chains, we refer the reader to [28, 29]. Here we will explain and make use of only those details which are directly relevant to our analysis. A Markov chain is usually represented as a directed graph (or network), with each Markovian system state pictured as a node and each possible system transition to an alternative set of states as a directed edge. Markov chains typically represent *stochastic* phenomena, where future states are only determined probabilistically by means of so-called *transition probabilities*. In spite of the simplicity of the Markov model, it reflects statistical patterns for the behavior of a complex system (such as weather) even though the detailed dynamical processes that lead to particular system states are ignored.

**Fig 2:**
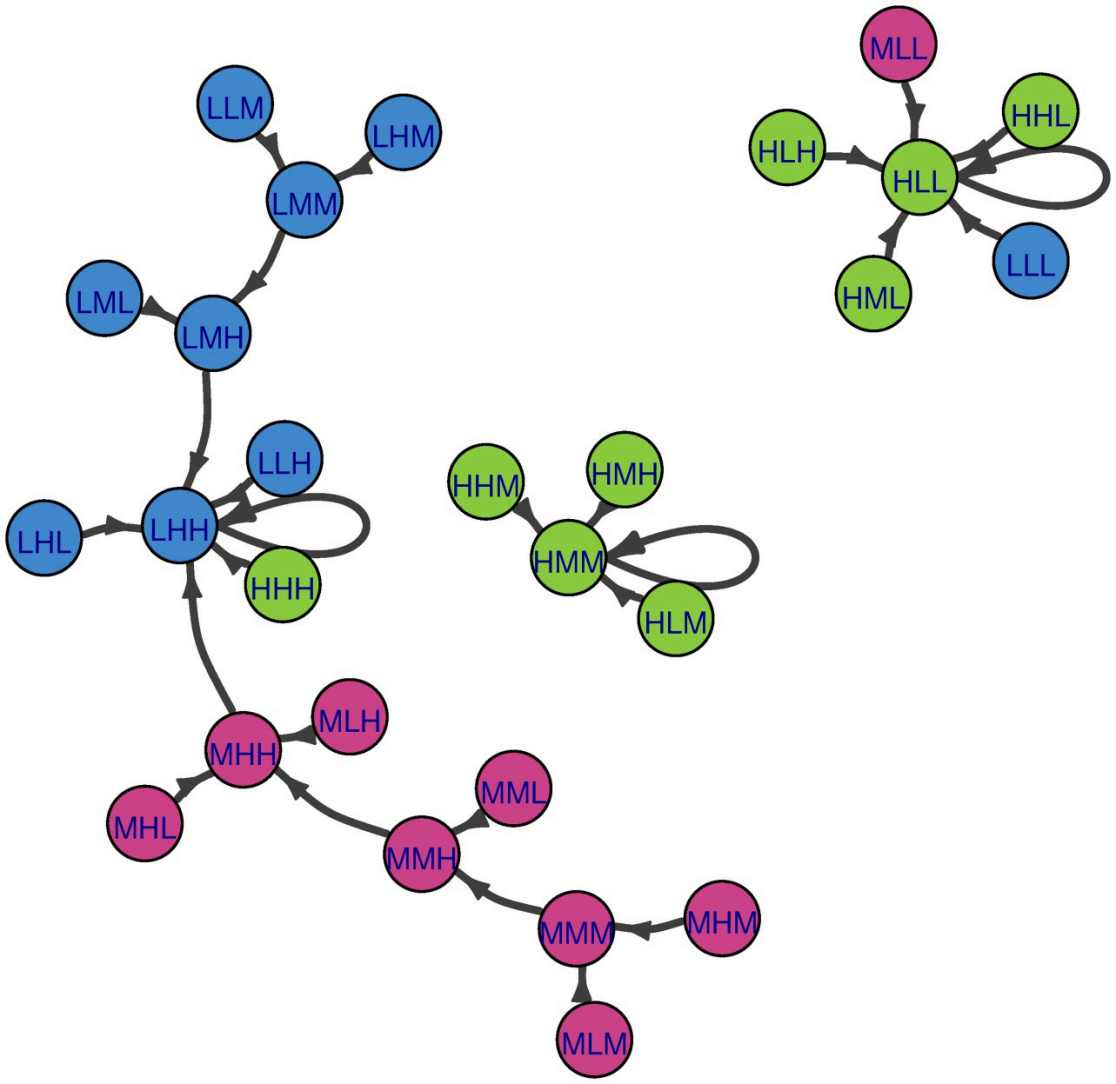
An example of a Markov chain with all transition probabilities equal to 1. Here we have taken the example scenario described in Fig 1 under the local succession (with a deterministic tiebreaking rule). All initial states filter down to one of three self-consistent states in a purely deterministic manner.

Classical CIB succession (as described previously in section 2.3) is not stochastic but still can be represented visually as a directed graph. If classical CIB succession were considered as a Markov chain, all transition probabilities would be equal to 1. This means that Markovian states (nodes) are CIB scenarios and Markovian transitions (directed edges) are CIB successions. An example of such a visualization is in Fig 2. This perspective yields a useful way to see alternative update *pathways* for a system created by a succession rule—as a network. It also suggests the possibility of modifying classical (deterministic) CIB succession with a stochastic approach. This will also allow all the mathematical tools associated with Markov chains to be brought to bear upon CIB analysis.

## 3. Calculation: Stochastic modification of CIB

Classical CIB succession can be visualized as a directed graph similar to a Markov chain, which allows us to consider system *evolution* under a variety of conditions, rather than merely the stable states of the system (the attractors). However, such directed graphs may give a simplistic view of the evolution of the system and suggest more certainty than is warranted. As Markov chains can be used to represent stochastic or uncertain phenomena, it is natural to wonder what might happen if, instead of a directed graph, we used a stochastic Markov chain—one where a single scenario can have multiple possible outcomes. In this section, we introduce the conceptual relationship between cross-impact balances and transition probabilities as well as four versions of stochastic updating rules: random descriptor, Boltzmann, logistic, and arctan. Finally, we discuss the application of entropy and entropy-production measures to enable a comparison of the different stochastic rules.

### 3.1. From cross-impact balances to transition probabilities

In CIB analysis, succession rules can be thought of as mappings that take in a cross-impact matrix and produce a list of all possible scenario-to-scenario transitions. Transitions between nodes in a Markov chain represent alternative system states according to some Markov rule. As discussed in section 2.3, CIB provides such succession rules for scenarios with weak internal consistency. Under classical CIB succession rules, each scenario has exactly one successor. That is, for a Markovian representation of classical CIB succession, the *transition probabilities* are 0 or 1: either scenario z’ is the only successor of scenario z, and the transition probability from z to z’ is 1, or it is not, and the transition probability is 0.

There is, however, a large variety of mappings that could be used to generate transition probabilities with values between 0 and 1—transitions with some chance of occurring, but no certainty. Regardless of the function used to calculate transition probabilities, it is important to note that we cannot expect to get an exact representation of system behavior from these equations. Some such more general mappings, defining a *stochastic Markov chain*, are described in the following subsections. Much like the classical deterministic CIB framework, the aim of a stochastic CIB framework is to synthesize the knowledge of experts in a variety of fields. However, results should still be treated as suggestive rather than predictive, as CIB modeling inherently represents systems that are not well understood. Results from applying these stochastic rules to the aforementioned Population-Income-Education example appear in section 4. The mappings used here were selected for their desirable mathematical properties and ease of calculation. Any reader who feels some other function is more appropriate to the system they are dealing with is encouraged to investigate. In choosing your own function, bear in mind:

- The transition probabilities must always be positive; negative probabilities are not mathematically valid.
- The transition probabilities must never exceed 1 –it is impossible to be more than 100% certain.
- The total transition rate from any given situation (including the transition rate back to itself for a self-loop) must add to precisely 1. This is because during any succession, some transition will always happen. For most functions, this can be achieved through normalization.

In this preliminary investigation, we have limited ourselves to stochastic succession rules that update at most one descriptor per succession (akin to the local succession rule described in section 2.3). This choice was made for simplicity. Succession formulae that deny this assumption may also be valid.

### 3.2. The random descriptor succession rule

The simplest of stochastic succession models we will refer to as *random descriptor* succession. During each step of the succession analysis, a single descriptor is selected at random and then updated to whatever descriptor state has the highest impact score with respect to the current scenario. In most cases, this means that for a system with three descriptors, each scenario may have up to three successors, and all transition probabilities are multiples of one third. A special case arises when there is a tie between descriptor states. In this case, updating to a randomly selected descriptor state may lead to transition probabilities finer than one third. Situations that are internally consistent will transition back to themselves with certainty (since they already have the maximum impact score for all descriptors), except for descriptor ties when transition to equally consistent scenarios is possible. For example, the scenario described in Fig 1 (Low population, High income per capita and Low educational attainment) has three possible successors. If education is selected to update, then the system will switch to the scenario “Low population, High income per capita, High educational attainment”. If income per capita is selected to update, it will switch to “Low population, Low income per capita, Low educational attainment”. If population is selected to update, it will remain in the current scenario.

Compared to classical “global” and “local” succession rules, random descriptor succession is far more likely to reach a consistent state, simply because it is less likely to become trapped in cyclic attractors (for example, the fifth time around a loop in a would-be cyclic attractor, the system has a fairly high probability of choosing a different path by chance). For example, if the small collection of nodes were A, B, C, rather than node succession being ABCABCABCABC (a cyclic attractor), we may encounter ABABCBABCCBABC. The concept of “basins of attraction” also becomes foggier under random descriptor succession, as a given starting situation may have the possibility of reaching multiple consistent states. This uncertainty can be seen either as a strength or a weakness of this succession rule; in the real world, it is perfectly normal to encounter several identical-looking scenarios that later find themselves in very different states. However, in calculating the probability of going one way or another using such rough metrics, these probabilities should not be mistaken for a predictive tool. They are merely a first approximation for a succession rule where the assumptions of determinism are relaxed. Nevertheless, random descriptor succession still mimics deterministic succession: Consistent scenarios are succeeded only by themselves. After a large number of successions, a system will eventually reach a steady state—either reaching a single consistent scenario, or a small collection of scenarios succeeded only by one another. This strong similarity indicates that random descriptor succession is only a slight modification of the classical deterministic succession rules.

### 3.3 Local stochastic succession rules: Boltzmann, logistic, arctan

As mentioned previously in section 2.3, local CIB succession is equivalent to updating the most severe descriptor inconsistency first. In the following modifications of local succession, we still update highly inconsistent descriptors first, but not with absolute certainty. Under stochastic versions of the local succession rule, we view descriptors with higher inconsistency as more likely to be changed than those with low inconsistency. For example, if a descriptor has an inconsistency measure of 5, it is reasonable to expect it to update before a descriptor with an inconsistency measure of 1.

During each succession, we assume that any descriptor could change to any state, but only one descriptor can change at a time. The driving force for such transitions is what we call the *impact gradient Δ*_*i*_ (*s*, *s*′; *z*) of two states s and s’ of the same descriptor *i*, given current scenario z. It is defined as the difference between the impact score of the target state s′ and that of the current state s: *Δ*_*i*_ (*s*, *s*′; *z*) = *Θ*_*i*_ (*s*′; *z*) − *Θ*_*i*_ (*s*; *z*). We then define the transition probability *T*_*i*_ (*z* → *z*′) between scenario z = [z_1_,z_2_,…] and scenario z′ = [z′_1_,z′_2_,…], where z_j_ = z′_j_ for all descriptors *j* except *i* by the formula

$$T_{i}\left(z\to z\prime \right)=\frac{f\left(\beta \Delta_{i}\left(z_{i},\;z\prime_{i};\;z\right)\right)}{f\left(0\right)+\sum_{j}^{}\sum_{s_{j}\neq z_{j}}f\left(\beta \Delta_{j}\left(z_{j},s_{j};z\right)\right)}$$ (3)

and the transition probability of a self-loop by

$$T_{i}\left(z\to z\right)=\frac{f\left(\beta 0\right)}{f\left(0\right)+\sum_{j}^{}\sum_{s_{j}\neq z_{j}}^{}f(\beta \Delta_{j}\left(z_{j},\;s_{j};\;z\right))}.$$ (4)

We deal with self-loops here as a special case so as to avoid self-loops being “over counted”. For the Population-Income-Education case, we could create a self-loop by transitioning population to its current state, or by transitioning education, or income. If we count all three of these possibilities, we end up multiplying the chances of a self-loop by the number of descriptors. Other authors may prefer not making this distinction, and we accept that any answer is in some sense arbitrary. Fortunately, many answers (including forbidding self-loops altogether) lead to reasonable dynamics. In Eqs (3) and (4), *f* is *a priori* some predetermined monotone increasing function, and β>0 is a positive number whose interpretation will be discussed below. The denominator ensures the normalization of the transition probabilities.

In this study, we considered three different functions (many others may be suggested):

1. The “Boltzmann” exponential, f(x) = e^x^.
2. The “arc-tangent” function, f(x) = π/2 + arctan(x).
3. The “logistic function”, f(x) = 1/(1+e^-x^).

**Fig 3:**
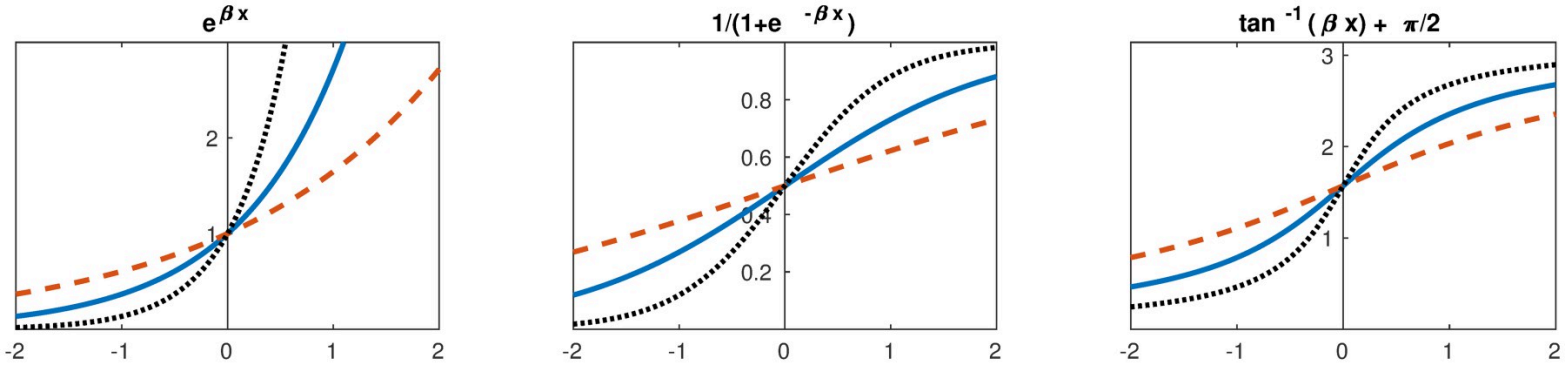
Plots of the three functions introduced in the text. From left to right: Boltzmann, arctan, and logistic, each for three values of β: β = 1(continuous), β = ½ (dashed), β = 2 (dotted).

In Fig 3, these three functions are plotted for comparison. Readers interested in all data sets and computational tools used in this article can find further information about data availability in Section 1 in S1 File.

The parameter β, which we call *stringency*, reflects how strongly scenario updating rules adhere to the cross-impact judgments provided by experts compared to random noise. In a very noisy system (small β, which corresponds with low confidence in expert judgments), our update is made approximately uniformly at random. In a highly deterministic system (large β, which corresponds with high confidence in expert judgments), the most inconsistent factor will be overwhelmingly likely to update, and our system will behave much as it would under the “local” succession rule of traditional deterministic CIB.

Local stochastic succession gives a positive number for all possible (single change) transitions. We note that it even gives positive numbers for transitions that are disfavored by the CIB analysis, although these are generally fairly small. Thus a system modeled using a local stochastic succession rule will never truly reach an attractor. This is because no matter how consistent a given situation is, there will always be some small probability of transitioning out of it. Every scenario is capable of reaching any other scenario in steps (where is the number of descriptors), although usually with extremely low probability. This dynamic is a sharp contrast to all other succession rules discussed so far, and is largely created by the small probability of states transitioning against the impact gradient Δ_i_.

### 3.4 Uncertainty and unpredictability (entropy and entropy production)

Because modifying CIB to include randomness leads to a certain degree of uncertainty in the “final state” of the system, conclusions from stochastic CIB analysis must be drawn in new ways. Although an end point for the system cannot be described with certainty, the probability of having the system be in any particular scenario after “a long time” can be stated. For instance, it could be said that, “If one waits long enough, the probability of the system being in the scenario with the outcomes ‘Low High High’ approaches 50%, while the probability of being in the scenario with the outcomes ‘High Low Low’ approaches 30%.” For local stochastic succession using any of the functions we have mentioned, any situation can eventually transition to any other situation. This leads to our initial scenario eventually becoming irrelevant as time goes on. For stochastic succession rules (such as local Boltzmann succession) where not all scenarios are connected by a transition path, we run into more difficulty, as the system will never completely forget where it started.

We refer to a collection of scenarios, along with a probability associated with each one, as a *forecast*, which we denote as *F*. A forecast can be used to compare multiple scenarios in a reasonable fashion. A forecast may refer to the probability distribution reached after an infinite number of successions—in Markov chain parlance, the *stationary distribution*—or it might be targeted at a particular time, for example, we might have the “forecast after three successions”. For the purpose of comparing notable scenarios, forecasts may uncover some inconsistent scenarios with high steady-state probability (for instance those that form part of a strong loop, perhaps with a large weighting of incoming edges). We may also find scenarios that are consistent under the original CIB rules, but with low steady-state probability (that is low probability according to our infinite time forecast). These may be scenarios that are only slightly more consistent than their ‘next-best neighbour’ or those with a small basin of attraction. We might think of these scenarios as ones which, while stable, may be less likely to be observed in real life, possibly due to a vulnerability to random noise, or simply because no series of events would be expected to result in their occurrence. It should be noted that under this formulation, specific forecasts depend on the value of the stringency parameter β and on the exact transition function *f* selected. The sensitivity of forecasts for noteworthy scenarios is discussed further in section 4.

Given a probability distribution on scenarios (for example a long-range forecast), it is useful to have some measure of how *uncertain* the forecast is. This can be done by simply looking at the probabilities, but for a more analytic expression, we calculate the *entropy* of the forecast:

$$H\left[F\right]=-\sum_{z}^{}\;F\left(z\right)\;log\;F\left(z\right).$$ (5)

This quantity, introduced in the context of information theory [18], is used in many fields of complex systems research to assess the “uncertainty”, “stochasticity”, or “spread” of a probability distribution. It can be viewed as a generalization of the notion of variance, ranging from H[F] = 0 for forecasts peaked on a single scenario to, where S_i_ is the number of states of descriptor *i*, for completely uncertain forecasts (in which each possible scenario is equally likely). For a system with 3 descriptors each having 3 states, there is a total of 27 possible states and a maximum entropy of 4.7548… (log_2_ 27). In other words, the entropy of a forecast provides an estimate of its definiteness: The lower the forecast entropy, the lower the number of scenarios with a high probability in the forecast. Readers interested in further discussion of entropy production are directed to Section 2 in S1 File.

## 4. Results

As discussed above in sections 2 and 3, by representing system successions as a Markov chain, it may be easier to study system *evolution*, rather than merely its stable states (system attractors). In the subsections below, we present visualizations and Markov chains for the simple Population-Income-Education system according to different rules for system succession. We used CIB algorithms to develop a software tool that would produce Markov chains of system succession. For systems that can be described with a sufficiently small number of descriptors and states (yielding on the order of 10^10^ possible scenarios or less), CIB identifies system attractors by assessing the internal consistency of each scenario possible. It should be noted that the Population-Income-Education system has only 27 (3^3^) possible scenarios, so full Markov chains of system succession contain 27 nodes.

**Fig 4:**
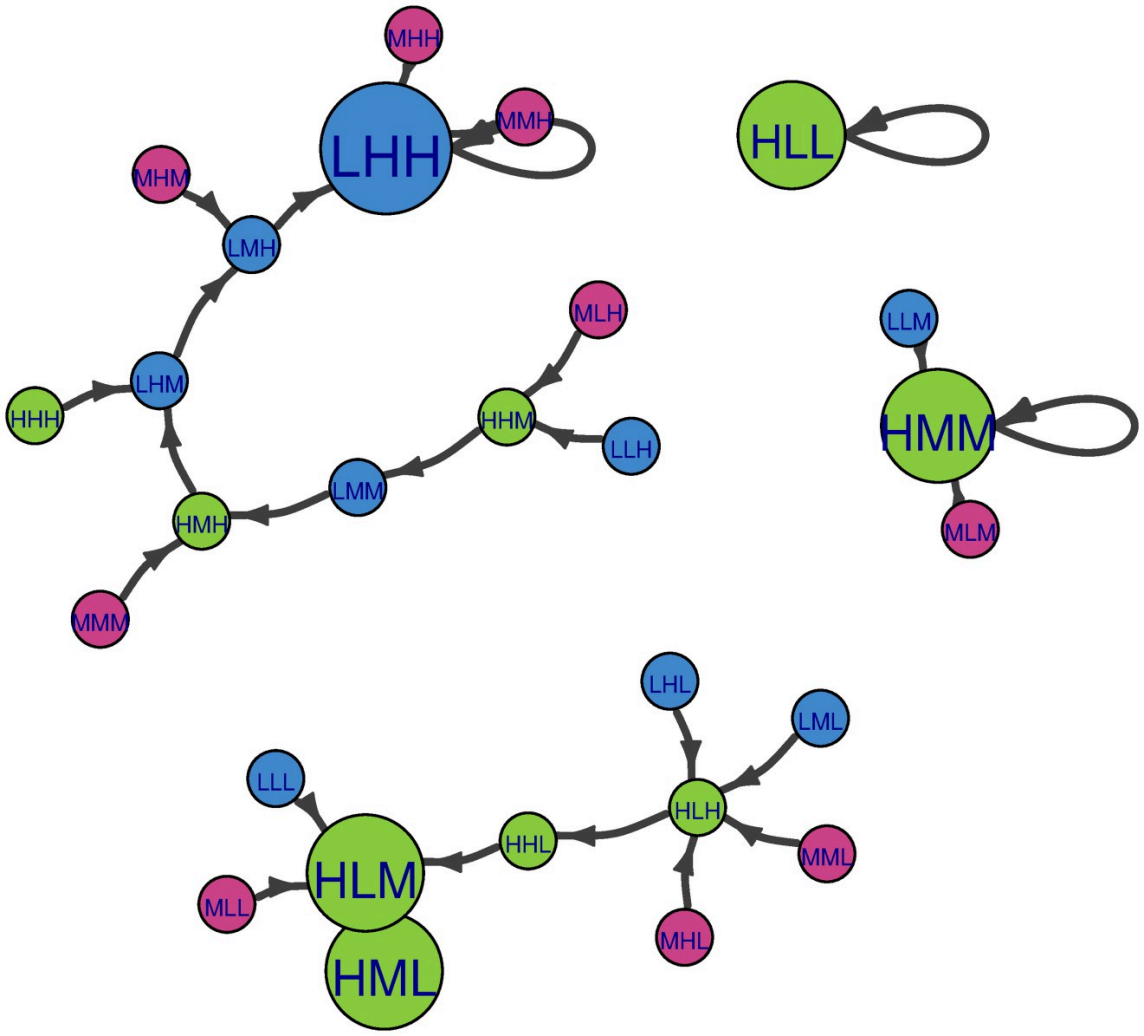
Markov chain of Population-Income-Education example with the global succession rule. L, M, H stands for Low, Medium, and High states respectively. The order of the states reflects the outcomes for the descriptors population, income per capita, and educational attainment respectively. Nodes are color-coded according to population (green-High; magenta-Medium; blue-Low). Transitions with probability 1 are black.

**Fig 5:**
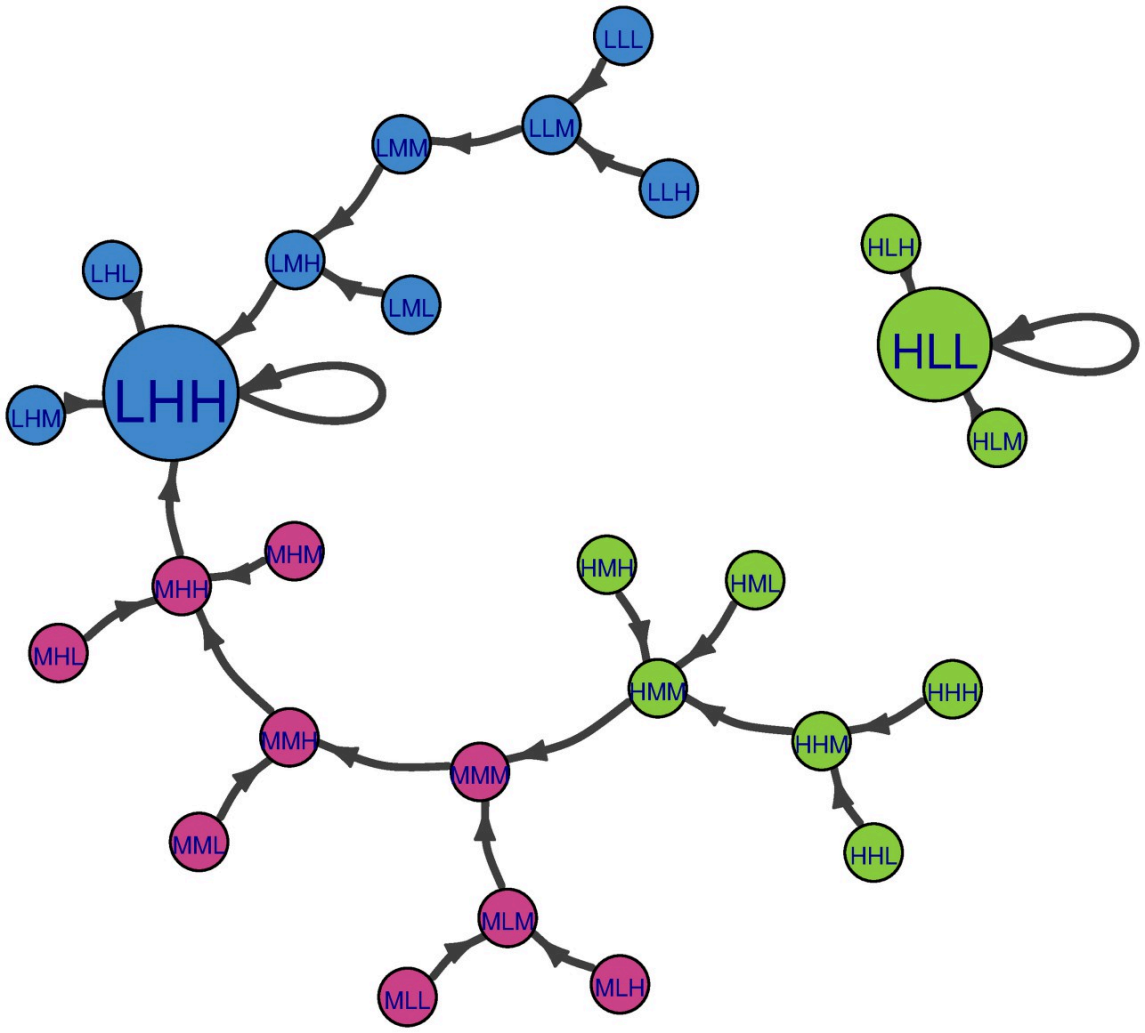
Markov chain of the Population-Income-Education example with the adiabatic succession rule. Color coding as in Fig 4.

**Fig 6:**
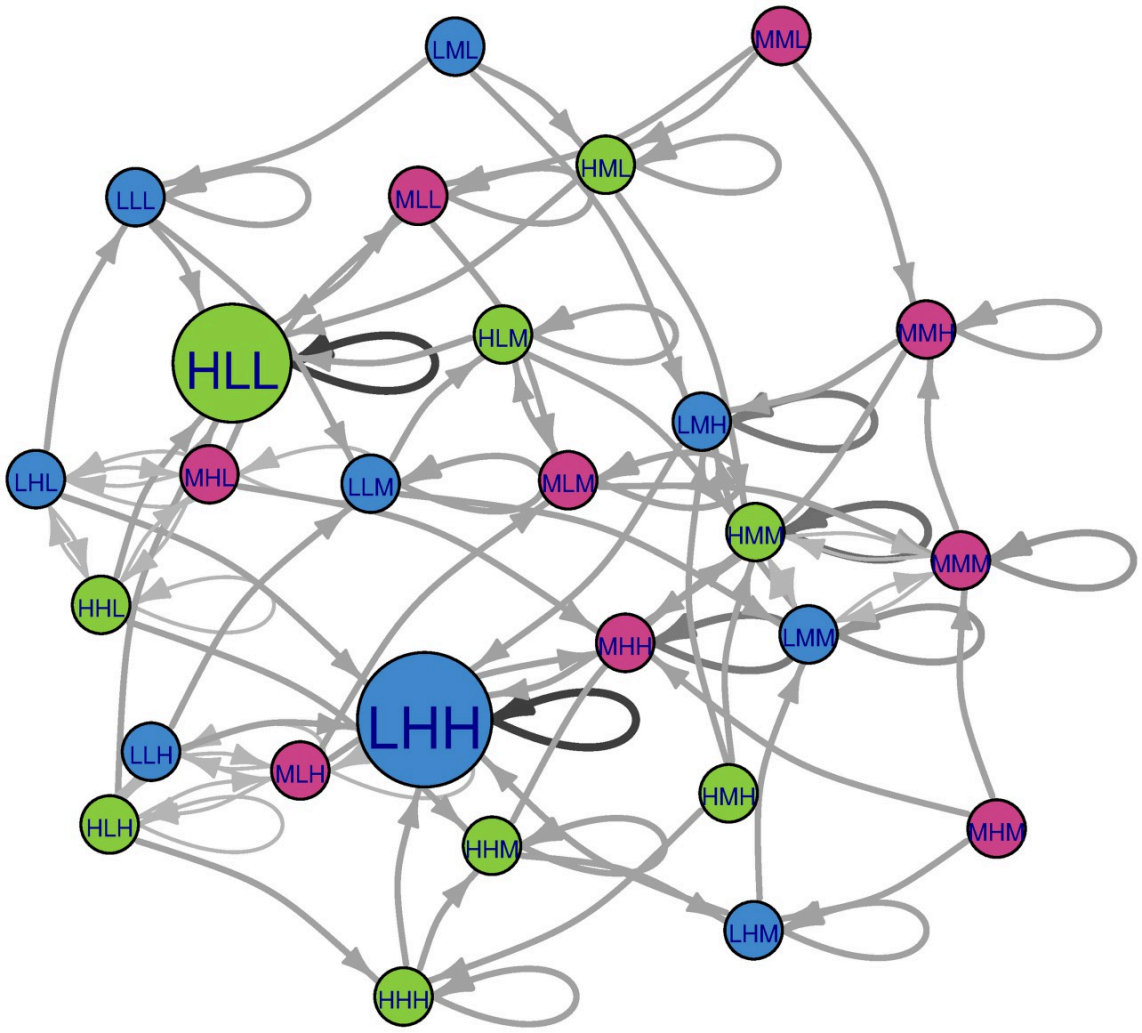
Markov chain of the Population-Income-Education example with the random descriptor succession rule. Color coding as in Fig 4. In cases where multiple states have the same impact score, succession is chosen at random amongst these states (grey edges). Node size is proportional to long term probability (assuming initial node selected uniformly at random). This is equivalent to eigenvector centrality.

**Fig 7:**
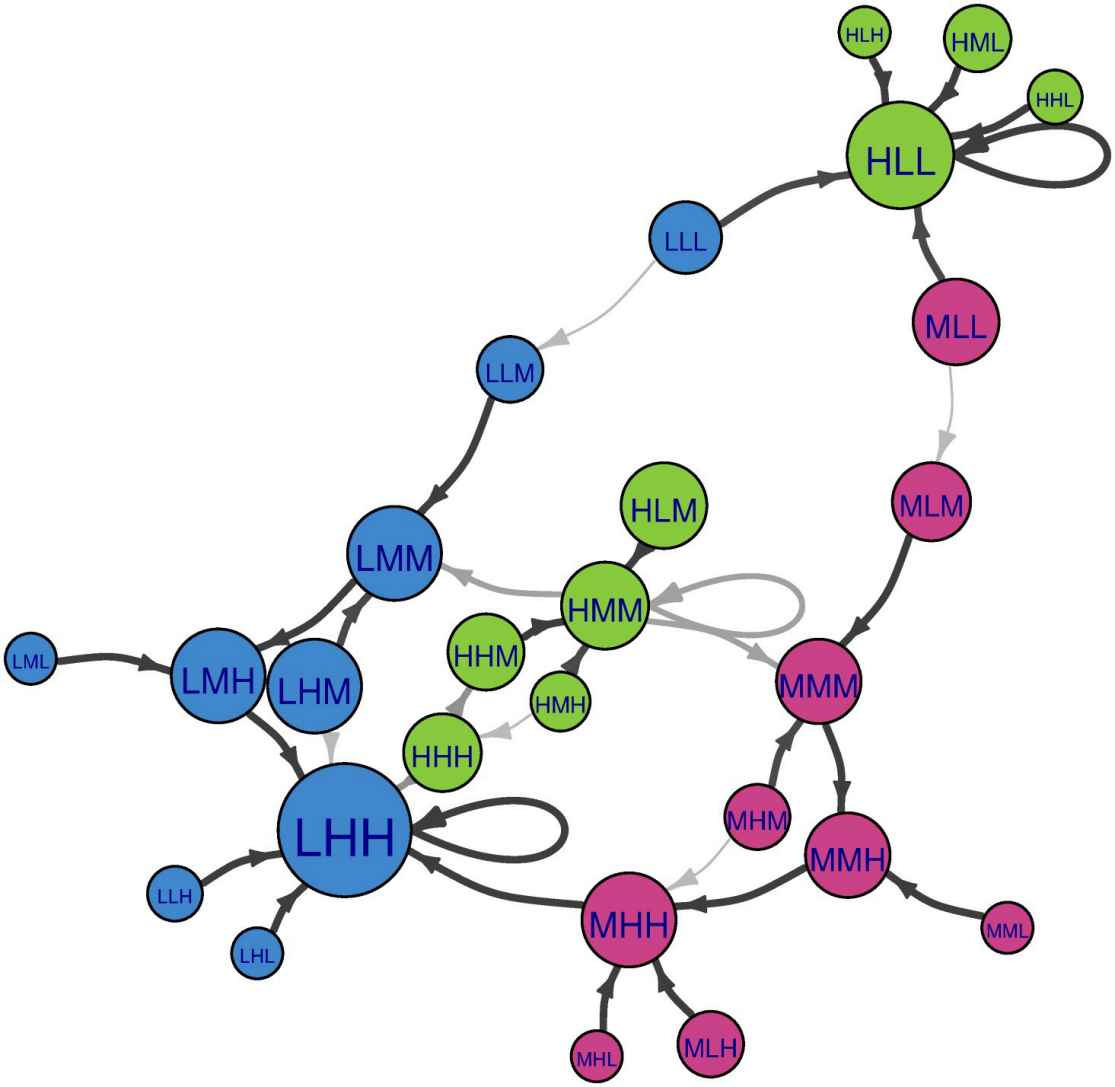
Markov chain of the Population-Income-Education example with the local Boltzmann succession rule (β = 1). Color coding as in Fig 6. Edges with weight less than 0.02 are not shown. Node size is proportional to the steady state probability of the node (long-term forecast). In this case, the initial state does not matter.

**Fig 8:**
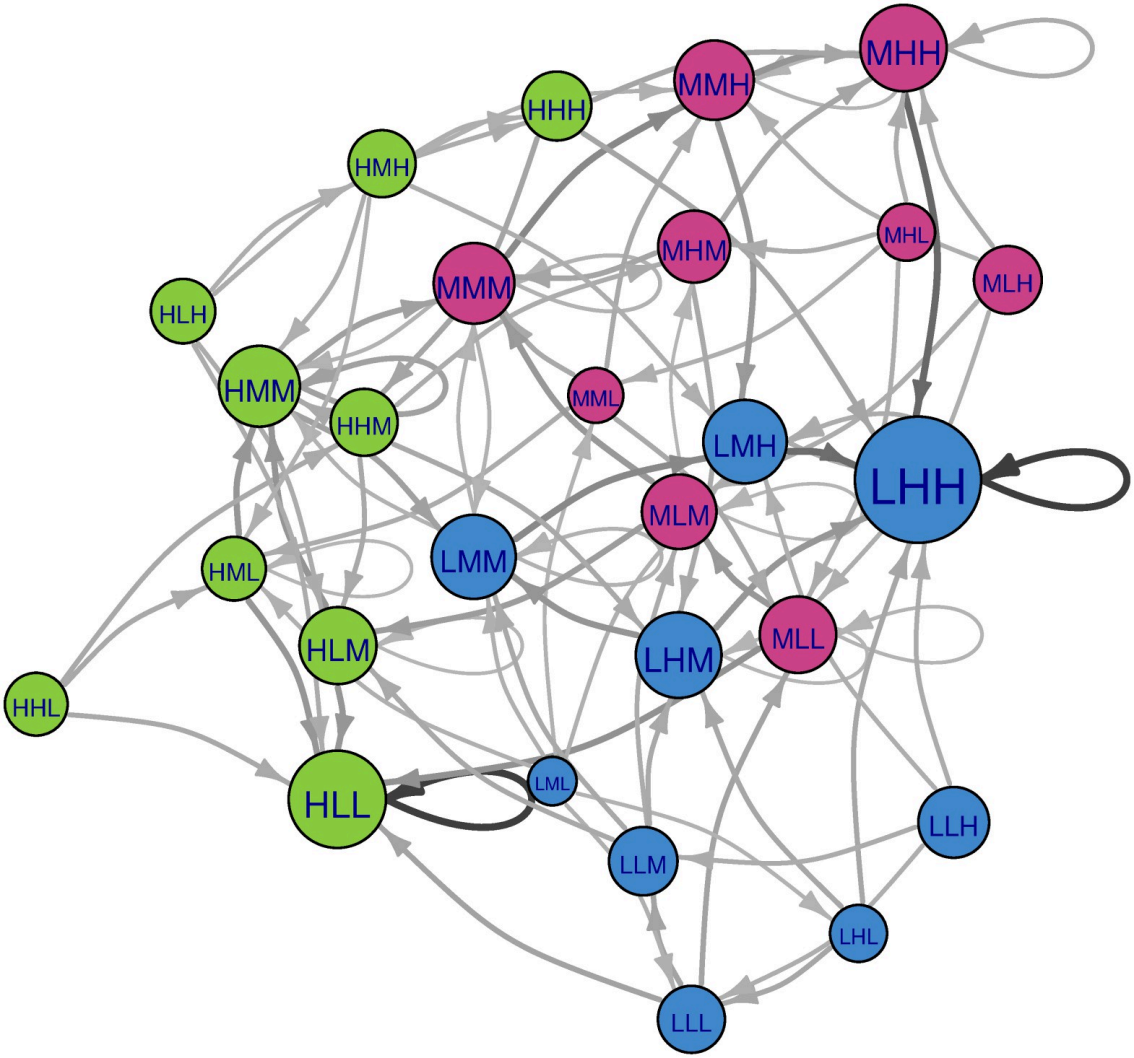
Markov chain of the Population-Income-Education example with the local logistic succession rule (beta = 1; shift = 1). Color coding as in Fig 6. To enhance clarity, edges with weights less than 0.1 are not shown in this figure. Node size is proportional to steady-state probability.

**Fig 9:**
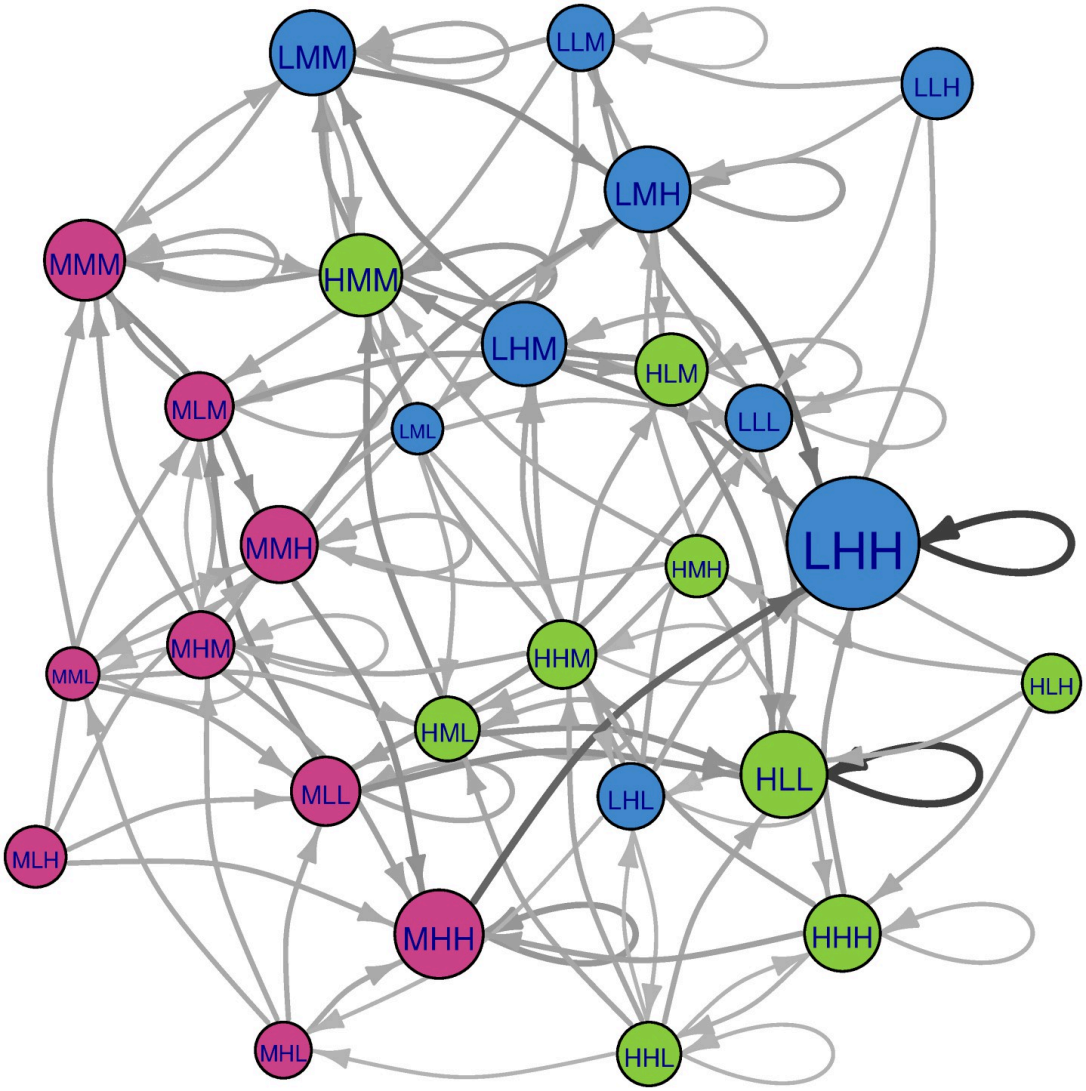
Markov chain of the Population-Income-Education example with the local arctan succession rule (beta = 1). Color coding as in Fig 6. For the sake of clarity, edges representing transitions with less than 10% probability are not shown.

For ease of comparison, all figures below (Figs 4–9) were produced using the R package igraph [30] and are presented with the same abbreviations, color coding, and motivation for node size. The abbreviations L, M, H stand for “Low”, “Medium”, and “High” states respectively. The order of the states pertains to outcomes for the descriptors population, income per capita, and educational attainment respectively. For ease of interpretation, color-coding is according to the state of population, where blue nodes pertain to Low population, magenta nodes to Medium population, and green nodes to High population. Probabilities shown on the edges are transition probabilities. Node size reflects the long-term (steady state) probability of ending up at a particular node assuming that our starting point is chosen at random. For networks where the measure of eigenvector centrality is meaningful, this is equivalent to steady-state probability. In section 4.1, we present visualizations of deterministic CIB (i.e., global succession and adiabatic succession rules) as Markov chains, and section 4.2 shows stochastic CIB Markov chains. Interpretations of the similarities and differences of the Markov chains are discussed in section 5.

### 4.1 Classical CIB succession: Global and “adiabatic”

As discussed in section 2.3, classical succession in CIB may be global or local. In the ScenarioWizard software package, there is a particular type of local succession called “adiabatic”, which we explain below. In this section, we present visualizations for deterministic global and adiabatic succession.

As a reminder, global succession updates all inconsistent descriptors simultaneously. Fig 4 shows a visualization of the entire Population-Income-Education system and succession pathways according to global succession. Under this succession rule, stable scenarios have no outward edges because they will not transition to alternative scenarios; instead, they have self-loops. Since succession is deterministic, each scenario has only one possible succession pathway (as shown in Fig 4, all edges have the same transition probability, which equals 1). For this example system, there are four stable scenarios corresponding to four basins of attraction. Some basins are larger than others. On the left, there is the “HMM” basin, where three scenarios converge. In the upper left, there is the small “HLL” basin, where only one scenario converges on this self-consistent state. In the upper right, there is the “LHH” basin. It could be argued that this is the largest basin, as 15 scenarios converge on LHH, and the large node size for LHH reflects this. At the bottom, there is the “HLM-HML” basin. The bidirectional link between HLM and HML indicates cyclic behavior as discussed above in section 2.3. It is also a sizable basin with eight scenarios as members. The larger node size for HML reflects this.

A classical succession rule that allows only one descriptor to change at a time is descriptor-ranked and called *adiabatic succession*. According to this rule, the analyst ranks each of the scenario descriptors in terms of “update speed”, and the internal consistency check for the scenario states begins with the ‘fastest’ descriptors before proceeding to slower ones. The analogy for this approach to succession comes from physical systems where some components move at relativistic speeds, e.g., molecular physics [31]. Approximations of overall system behaviour can be made by treating the subsystems (i.e., faster vs. slower) separately. For the example Population-Income-Education system, one could imagine that the global population descriptor might update the slowest. At any given succession step, whichever inconsistent descriptor is ranked to update the fastest is updated first. The fast descriptor would then be updated to whichever descriptor state has the highest impact score before proceeding to descriptors that update more slowly. This form of succession analysis gives some hint of a temporal component, but like all the deterministic succession rules described, it is not interpreted to give a realistic representation of chronological system behavior. Fig 5 shows a visualization of succession pathways according to adiabatic succession. As was the case for global succession discussed previously, stable scenarios have no outward edges because they will not transition to alternative scenarios; instead, they have self-loops. Additionally, each scenario has only one possible succession pathway.

Under adiabatic succession, there are three basins of attraction for the system. First, it should be noted that the cyclic attractor present under global succession is not present under adiabatic succession. In the upper left, the HMM basin is present again, where four scenarios converge. In the lower left, the HLL basin is present again and is larger containing six scenarios. At right, there is the large LHH basin again with 17 scenarios as members, which corresponds with the large node size for LHH.

### 4.2 Stochastic succession: Random descriptor, Boltzmann, logistic, arctan

As discussed in sections 3.2 and 3.3, we considered four stochastic succession rules. The first is the random descriptor rule discussed in section 3.2. The other three stochastic succession rules are discussed in section 3.3. and are based on the mathematical functions of the exponential curve (Boltzmann), the logistic curve, and arc-tangent. Here we present stochastic Markov chains for each of these succession rules. As mentioned previously, none of the results here should be viewed in any way as a precise forecast, but instead as a (mathematically aided) thought experiment. Hence, it may be useful to say “Scenario LHH appears stable” but would be ill advised to claim “Scenario HLL has probability 0.473”.

As discussed in section 3.2, the random descriptor succession rule is a type of local succession, where any descriptor is chosen at random and updated regardless of whether the descriptor is inconsistent or not. Fig 6 shows a visualization of succession pathways according to such succession. As would be the case for the deterministic succession rules discussed previously in section 4.1, self-consistent scenarios should have no outward edges because (regardless of which descriptor updates) they will only ever transition back to themselves. Now that the Markov chain is stochastic, each scenario has multiple succession pathways. Results from random descriptor succession are interesting in that all outgoing edges (with the exception of self loops for attractors) have a probability at either 0.33 or 0.2 due to the random nature of the method. However, it is important to note that not all nodes have self-loops. For scenarios without self-loops, this means that remaining at their particular conditions are not an option, which in CIB parlance, corresponds with high internal *inconsistency* for the scenario. The nodes with the largest eigenvector centrality in the system are LHH followed by HLL. This means that many scenarios may transition to LHH and HLL. The steady-state probability of all other nodes is zero. We find the uncertainty of our long-term forecasts (measured by entropy as explained in section 3.4) to be 0.79.

As discussed in section 3.3, local Boltzmann succession is a type of local succession, where highly inconsistent descriptors are still updated first, but not with absolute certainty. Which descriptor will be updated is determined probabilistically, where descriptor inconsistency (the impact gradient, Δ_i_) and stringency (β) are important factors. Fig 7 shows a visualization of succession pathways according to local Boltzmann succession. It is important to note that not all transition edges are illustrated—edges with transitions with sufficiently low probability (less than 0.02) are suppressed. Highly stable scenarios (such as LHH) only transition outwards with negligible probability (such edges are ignored in our diagram), while moderately stable scenarios (such as HMM) may have both self-loops and outward edges with non-negligible weight. Unlike the random descriptor succession rule, stochastic succession gives high probability not just to perfectly consistent scenarios, but also moderate probability to their neighbors, highlighting both scenarios and clusters of interest.

In Fig 7, the HLL cluster at right has a very weak connection to the LHH cluster at left (two edges with probability 0.05). Nevertheless, it is possible for scenarios in the HLL basin to find their way to another basin. Scenarios in the HLL cluster transition to HLL with high probability, and can only transition out with negligible probability. However, in the LHH cluster, the transient path is more complicated, primarily because of the presence of another attractor, HMM. Most of the successions for the system overall are highly predictable, but HMM introduces pathway uncertainties. This is because the HMM state is consistent with respect to education and income, and neutral (having a zero gradient) with respect to population. This effectively leaves future population levels up to chance. From HMM we encounter two distinct paths leading to LHH—one path through MMM or another through LMM. Compared to the other two consistent scenarios (HLL and LHH), the self-link of scenario HMM is not 1. As a consequence, the steady-state probability of HMM is significantly lower than the other consistent scenarios. Here we see one significant advantage of our stochastic perspective; while deterministic CIB methods view scenarios as either “consistent” or “inconsistent”, a stochastic approach allows us to distinguish between “more likely” or “less likely”. We are also able to clearly identify the existence of potentially critical ’branch points’- scenarios whose outcome has a disproportionately large impact on future trajectories. Such scenarios may be of interest to modelers and researchers, who might (for example) wish to supplement their understanding by investigating the outcomes of historical situations matching the highlighted scenario.

Under the Boltzmann update rule with β = 1, we calculate the long-term uncertainty of our forecasts to be 0.098 –significantly less than that of the random descriptor succession rule. This is the result of the LHH state becoming almost inevitable; the eigenvector centrality (steady state probability) for LHH is 1 while that of all other nodes are less than 0.002.

Section 3.3 also described another version of local stochastic succession called local logistic succession, and Fig 8 shows a visualization of succession pathways according to this succession rule. Compared to Boltzmann succession, a far greater probability for self-loops amongst unstable scenarios can now be observed. Referring back to Fig 3, this is because the logistic function treats all positive Δ virtually the same, while the Boltzmann function amplifies small discrepancies between positive values for Δ. Because of this, the system successions will not be forced along the path of steepest impact gradient as strongly as for the Boltzmann succession rule. In Fig 8, the local logistic Markov chain shows greater uncertainty in the pathways between scenarios. Rather than having transition probabilities close to 1, transition probabilities are generally smaller. Nevertheless, two stable attractors (HLL at lower left and LHH at right) both display self-loops with probability 0.98.

For many “intermediate” scenarios, in contrast, we observe multiple pathways with transition probabilities in a similar range (0.20–0.30). This results in a larger number of significant edges for the Markov chain. In addition, there are bidirectional links (cyclic behavior) between scenarios. For example, for the edge between LMM and MMM (toward the middle of the graph, left of center), these scenarios can transit back and forth with equal probability (0.23). The eigenvector centrality for LHH is close to 1 while that of all other nodes are less than 0.02. In spite of the high steady-state probability for the LHH node, the uncertainty in our long term forecast for β = 1 is significantly greater than what we had for the Boltzmann update rule, with a value just over 0.56. However, its uncertainty measure remains less than that for random succession.

The final version of local stochastic succession discussed in section 3.3 is local arctangent succession, where the arctan function is applied in the succession rule. Fig 9 shows a visualization of succession pathways according to local arctan succession. Stable attractors other than LHH become difficult to identify under this succession rule. This reflects particular mathematical properties of the arctan function, namely that the transition probability penalty associated with having a negative impact gradient is much lighter than it would be for the logistic or exponential functions. This allows “leakage” from the stable attractors, as disfavored transitions are only somewhat penalized. This means that scenarios tend to update to alternative scenarios regardless of their levels of self-consistency. Additionally, this means that the arctan succession rule generates many bidirectional links (cyclic behavior) between nodes. Pathway probabilities away from inconsistent scenarios are higher (see nodes at the periphery of the network such as LML, LLH, MHL), but the weights for self-loops in general are lower. The eigenvector centrality for LHH is 1 while that of all other nodes are less than 0.25. This significant increase in the steady-state probabilities of non-LHH nodes corresponds to a jump in the uncertainty of our long-range forecast of the system, with entropy jumping to 3.57.

## 5. Discussion

In this section, the findings from section 4 are discussed and interpreted. As discussed in the Introduction, the primary motivation of this project was to further improve CIB analysis, which takes a deterministic approach to system succession. Thus to determine whether our stochastic methodological modifications are useful in this regard, it is necessary to compare the Markov chains of the Population-Income-Education system under different succession rules. We do this in section 5.1. In section 5.2, we consider whether the robust findings across the different succession rules are suggestive for ongoing research in the field of demography. In section 5.3, we comment on how the tools developed for this study could be applied to cases of more complex systems.

### 5.1 System attractors and other notable scenarios by succession rule

In Table 1, we summarize notable scenarios (Markov chain nodes) identified by the five succession rules featured in section 4: global, adiabatic, local Boltzmann, local logistic, and local arctan. We identified some findings that were common across all succession rules as well as some findings that were sensitive to the choice of the succession rule. Differences in the findings suggest particular tradeoffs among succession rules.

There are two common findings across all succession rules, whether they are deterministic or stochastic. First, the scenarios LHH and HLL are found to be attractors, and for all succession rules that identify attractors with probabilities of self-looping greater than 0.95 (henceforth called ‘stable’ attractors), both of these are stable. Moreover, deterministic CIB finds LHH to be the attractor with the largest basin of attraction, while all stochastic CIB rules identify the LHH node as the set of states that the system will settle upon after a long time. This consensus between modelling would appear to be strongly indicative of the importance of these states given the cross-impact judgments in this particular model.

Second, the scenario HMM is found to be an unstable attractor. Even the global deterministic case suggests that HMM is a weaker attractor, as it has the lowest *total impact score* [8] among non-cyclic attractors. The total impact score represents the strength of the self-consistency of a scenario, and when it is low, it means that it would be easier to push the system away from the attractor (compared to other attractors). Higher than average uncertainty in where the HMM state is pushed *to* is suggestive of this scenario being of further research interest. More detailed expertise and analysis may be able to clarify the conditions under which HMM would be pushed to take different paths to the stronger attractors of LHH (i.e., decreased population, increased income per capita) or alternatively HLL (retaining high population, decreased income per capita). A review of demographic literature and how its findings jibe with our Markov chain models is discussed in section 5.2.

In contrast, there are two findings that are sensitive to the choice of succession rule. First, the cyclic attractor HLM ↔ HML is found only under global succession. For all other update rules, no more than one descriptor can be updated on any given timestep, meaning that direct transition between these two states is impossible. This suggests that this cyclic attractor is an artifact of updating all descriptors simultaneously. Second, MHH is simply an inconsistent scenario under the global succession rule, while it is much more interesting under alternative succession rules. All local succession rules (including the deterministic adiabatic rule) show MHH to have moderate or high levels of transient traffic. Under the random descriptor, logistic, and arctan succession rules, MHH is an unstable attractor but equally or more stable than HMM (which was identified as an attractor under deterministic succession) due to its higher probability for self-looping. These findings suggest a tradeoff with the global succession rule, where potentially useful information about transient system behavior may be lost. In section 5.2, we take a closer look at the value of this information on transient system behavior in light of a recent review of demographic literature.

Across all succession rules, there are tradeoffs. As discussed above, while global succession may quickly converge on system attractors, its simultaneous updating of all inconsistent descriptors may produce artifacts. Additionally, potentially useful information about transient system behavior may be lost. In our study of this three-descriptor system with β = 1, we found that results from the local Boltzmann succession rule correspond nicely with deterministic results yet recover additional transient information. To be more specific, pathways that potentially bridge basins of attraction can be seen with stochastic succession. Local logistic succession may be a further improvement over Boltzmann, as information about stable attractors is retained while more unstable attractors, such as MHH, come to the fore. However, uncertainty remains much higher under logistic succession compared to Boltzmann. Local arctan succession has higher uncertainty still, and information about stable attractors is lost. Based on this investigation, it would appear that which succession rule is most appropriate will depend on the uncertainties inherent in the system: in systems where experts claim some moderate degree of certainty, Boltzmann succession may be most appropriate. In those systems with significantly higher degrees of uncertainty, logistic succession would appear more appropriate.

### 5.2 Suggestive findings of the Population-Income-Education example

Any modelling effort, including the application of CIB, aims to reveal something about the world. As discussed above, all succession rules identify the LHH node as the most significant attractor of the Population-Income-Education model. This finding is a reflection of one of the most empirically established theories in the social sciences, the demographic transition [32]. The theory refers to a shift in societal conditions from high birth and death rates with minimal economic development and education to low birth and death rates with improved economic development and education. However, there are still countries that have not completed their demographic transitions and still have high fertility rates, many of which are also low-income with low levels of educational attainment (i.e., epitomizing the HLL attractor). In this section, we focus on the information afforded by stochastic CIB rules on transient system behavior and consider whether the simple Population-Income-Education model hints at strategies for how such countries might escape this fate. We discuss these findings in light of ongoing research in human demography. Jiang [33] provides an especially relevant study for this purpose, as he reviewed the demographic literature on the pairwise relationships between population, education, economic growth (measured by the rate of change for GDP per capita), and urbanization. He did this for the purpose of assessing global scenarios called the Shared Socio-economic Pathways (SSPs), which entered the literature around the same time for the purpose of climate change research. For comparison with the results of our simple model, we look only at his discussion of relationships between population, education, and economic growth.

As noted in section 2.1, our CIB model is based on a subset of judgments collected by Schweizer & O’Neill [13], who contributed to SSP development. They explained that SSPs should be considered aggregated and averaged trends for each variable at the global level. However, Jiang explains that demographers have concluded that the interactions between these variables differ across stages of economic development. Thus it’s important to consider *rates of change* to the variables rather than the levels of the variables themselves. For this reason, Jiang parses the literature of bidirectional effects as being applicable to low-, medium-, and high-income countries. This suggests that the mapping of our model results to Jiang’s discussion of demographic literature be done with care. In Section 3 in S1 File, we review the meanings of the states of each variable from Schweizer & O’Neill [13] as well as the verbiage employed by Jiang [33] in his review. We find strong agreement between the concepts discussed, which provides confidence for the external validity of our conclusions.

Results from deterministic CIB provide the clue that demographic outcomes for human societies have 3–4 attractors, each with basins of differing size (Figs 4 and 5). However, these succession rules do not provide information for how a country currently in the HLL or HMM basins might kick-start (or sustain) a demographic transition to the LHH basin. Below we consider the information of transient system behavior provided by non-random stochastic succession rules and their insights for paths that traverse basins.

The first finding to note is that HLL is a formidable attractor, even under stochastic CIB. Circumstances that are more similar to HLL (especially sharing two out of three states with HLL) have high probability gradients back to HLL under all stochastic CIB rules. This suggests that the demographic transition may be unlikely to be sustained through piecemeal approaches to improving either income growth or educational attainment alone. This lends credibility to the notion that there is a “demographic trap” (see e.g. [34]).

Second, under the Boltzmann succession rule (Fig 7), some network structures are the same as adiabatic CIB, namely the presence of the HLL and HMM basins as well as the MHH Markov chain (cluster of magenta nodes in Fig 5). Agreement in the network structures between the Boltzmann and adiabatic CIB rules provides some confidence that the structures resemble real phenomena rather than being artifacts. Boltzmann succession provides the clue that there may be two paths away from the HLL basin, each with small transition probabilities (*T* = 0.05). These are via the nodes MLL and LLL, both of which ‘escape’ to nodes with higher educational attainment (transitions to MLM and LLM respectively). These paths indicate two possible mechanisms. One is that once fertility rates decrease, this opens up the possibility for changes to educational attainment. Jiang explained there is some support for this view in the demographic literature (with both agreement across studies and quality of evidence rated as medium). Declining population growth (i.e., lower fertility) incentivizes educational attainment because uneducated children come to be seen by their parents (and perhaps society) more as potential liabilities and less as assets for labor (Weiner 1991 in [33]). The second possible mechanism is that changes to educational attainment happen first, thereby initiating decreases in fertility [35] that pull the system toward the LHH basin (from the H population state to M or L). For the latter mechanism, Jiang rated the agreement across studies as high and the evidence as medium.

Under Boltzmann succession, it should also be noted that the HMM node has equal transition probabilities (each *T* = 0.33) for remaining stable or transitioning further via MMM to the MHH Markov chain, which ‘empties’ to the LHH basin. Another possible path away from HMM is via LMM, which is part of a Markov chain to the LHH basin. This suggests that if an HLL society can ‘jump’ to the HMM state—such as through prioritizing mass education for political reasons (such cases were uncovered in Jiang’s review)—chances are good that it will continue along some path to the LHH attractor. However, it should be noted that the HMM node does not have a reliable connection to the HLL basin (*T* ≤ 0.02). This means it is not as easy for the model to jump to HMM from HLL as it is to transition to MLL or LLL, which may be a model artifact of needing to change the state of only one descriptor (Population goes from H to M or L) rather than two descriptors (Economic growth and Educational attainment must simultaneously change from LL to MM).

The local logistic succession rule (Fig 8) refines one of the insights provided by Boltzmann succession. Under the logistic rule, higher transition probabilities away from HLL (*T* > 0.3) correspond to portions of the MHH and LMM Markov chains that were also seen under Boltzmann succession. Logistic succession provides the additional clue that the path away from the HLL basin is most likely via MLL (*T*(MLL→MLM) = 0.35). It is intuitive that the transition probability from the high population state to the medium state should be more likely than the bigger jump from high population to a low state (in contrast, recall that these transitions were equally possible under Boltzmann succession). However, Jiang notes that demographic research shows that improved educational attainment induces strong negative influences on fertility rates, both at the early and latter stages of the demographic transition. Perhaps transition probabilities to the medium population state (i.e., a lower total fertility rate, but equal to or above replacement) and to the low population state (fertility below the replacement rate) may be nearly equal after all.

Third, local arctan succession (Fig 9) provides further information. For escaping the HLL basin, there are multiple possibilities for taking a ‘first step’ away, each with *T* = 0.16. However, only a few of these steps have higher transition probabilities (*T* ≥ 0.2) to subsequent steps leaving the HLL basin. Possible mechanisms are to increase income growth first (via HML) or to decrease fertility rate first (via MLL or LLL). When HML transitions away from HLL, it does so via HMM (*T*(HML→HMM) = 0.4). When LLL transitions away from HLL, it does so via LLM (*T*(LLL→LLM) = 0.25), and when MLL transitions away from HLL, it does so via MLM (*T*(MLL→MLM) = 0.2). These key ‘first steps away’ from HLL also have differing transition probabilities for slipping back to the HLL attractor (*T*(HML→HLL) = 0.4, *T*(LLL→HLL) = 0.25, and *T*(MLL→HLL) = 0.2 respectively).

The arctan findings are noteworthy for three reasons. First, arctan succession identified all the transition paths suggested by Boltzmann succession. This further raises confidence that the transition paths may be meaningful rather than artifacts. Second, arctan succession identified paths from HLL to HMM that were tenuous (or non-existent) under Boltzmann succession. This provides the ability to inspect the feasibility of such paths. Third, the information provided by arctan succession suggests that sustained decreases in fertility are more likely to succeed if efforts to enhance educational attainment occur while the economy grows as well.

We arrive at the third finding by focusing on the path away from HLL via HML, as its transition probabilities along the path differ the most compared to random chance. There are 6 nodes that HLL could transition to (including remaining in a self-loop); nodes that are not the HLL self-loop are one degree of separation from HLL. HLL is equally likely to remain stable or to transition to any of these one-degree nodes (i.e., *T* = 0.16; note that HLL would not transition to HLH, which is simply an internally inconsistent scenario). This suggests that when Economic growth and Educational attainment are both low, it is just as difficult to sustain economic growth (transitioning from low to medium income; *T*(HLL→HML) = 0.16) as it is to increase educational attainment (transitioning from low to medium educational attainment; *T*(HLL → HLM) = 0.16). Nodes that are two degrees of separation from HLL (i.e., directly connected to a neighbor of HLL) have lower transition probabilities away from the HLL attractor (*T* ≤ 0.25), with the exception being the HML node, which has *T*(HML → HMM) = 0.4.

For the HML scenario, there are bidirectional transition probabilities. When educational attainment remains low while efforts are made to increase income, it is highly likely that conditions will return to the HLL attractor (*T*(HML → HLL) = 0.4). However, it could be just as likely that HML will continue transitioning to HMM (*T*(HML → HMM) = 0.4). From the demographic literature, Jiang provides an explanation, which is that wealthier households are more willing to accept the opportunity cost of education (i.e., temporarily forego income from children’s labor).

Finally, in contrast to Boltzmann succession, the arctan rule finds a clearer path away from HLL to the HMM attractor (via HML) rather than via LLL or MLL. The latter paths are more stochastic, with transition probabilities away from (and returning to) the HLL attractor being only slightly better than random chance (0.16 ≤ *T* ≤ 0.25). Jiang’s literature review provides context for why the paths through LLL and MLL scenarios may be less certain for leaving the HLL basin: Lack of income, whether considered at the societal or household levels, is largely considered the most significant barrier to educational attainment. At the societal level, mass education is a substantial public investment that is difficult to initiate and maintain. In fact, in poor countries where the provision of basic education is expensive, enrollment in primary school can be negatively related to income growth. This may be because, in poor countries, child labor often makes important contributions to household income; thus parents find little incentive to send children to school.

Across the various stochastic succession rules, the information provided on transient system behavior jibes with conclusions drawn in the field of demography. The most useful information was provided by the local Boltzmann and arctan succession rules. As discussed in section 5.1, the local arctan rule loses information about stable system attractors; however, it appears to produce the most detailed information about likely transition paths across basins. For the simple Population-Income-Education model, local arctan succession and Boltzmann succession agreed that HMM is a key node for escaping the HLL basin. This suggests that the Boltzmann and arctan succession rules should be used in tandem to first identify and then interpret both stability landscapes of system attractors [36] and various transition paths that are meaningful.

### 5.3 Challenges with more complex systems

This study features a three-descriptor system for illustrative purposes. The concepts and tools developed in this project can also be applied to more complex systems with more descriptors and states. Section 4 in S1 File contains a visualization of a Markov chain applying the local Boltzmann succession rule to a six-descriptor example (where each descriptor has 2–3 states, yielding 486 possible scenarios). However, with a higher number of network nodes, it becomes more difficult to identify visually self-loops, basins of attraction, and high-probability succession pathways.

## 6. Conclusions

This paper demonstrates how a full set of scenarios can be explored comprehensively, and how such exploration can reveal both stable, internally consistent scenarios (system attractors), and unstable but interesting transition scenarios as well as transition paths between them. Through this study, we addressed five research objectives. First, to investigate scenarios with weak internal consistency and their succession to system attractors, we found the concept of a Markov chain useful. We then developed a software tool based on the Markovian concept and investigated the case of a simple Population-Income-Education system with three variables (or CIB descriptors), each with three possible states. The simple system has a total of 27 possible scenarios.

Second, we modified classical deterministic CIB succession rules to consider four cases of stochastic CIB succession: random descriptor, local Boltzmann, local logistic, and local arctan. In our systems-level comparison of the Markov chains produced by these stochastic succession rules, as well as two classical deterministic rules (global and adiabatic), we found that stochastic succession reveals additional information about system behavior. This is because classical global succession may arrive at cyclic attractors that are difficult to justify when compared to local succession. Additionally, the global succession algorithm may trade information about the transient behavior of a system for finding system attractors expeditiously. In contrast, local succession brings information about transition pathways to the fore. Scenarios with ‘high traffic’ in transient pathways (that is, scenarios that are transitioned to by many other scenarios, even if they are not stable attractors themselves) become most obvious with the stochastic succession rules of local logistic or arctan.

Third, in our scenario-level comparison of the Markov chains, we found that many noteworthy scenarios for the system, such as its basins of attraction, are mostly unaffected by the choice of succession rule. If one takes the view that the primary strength of CIB is its identification of stable system attractors, this strength may be relatively insensitive to the choice of succession rule. However, this was not the case for the local arctan succession rule, as information about stable attractors was lost. We also found that a cyclic attractor present under global succession was not present for other succession rules. In contrast, we found a notable scenario, MHH, which has moderate- to high-traffic under local deterministic and stochastic succession (and is an unstable attractor under local logistic succession). However, under global succession, it is an uninteresting low-traffic, inconsistent scenario. These scenario-level differences in the findings underscore the tradeoffs that come with selecting particular succession rules.

Fourth, we used the concepts of entropy and entropy production to develop measures for comparing the uncertainty (entropy) and unpredictability (entropy production) of forecasts of system behavior under different succession rules for CIB. We found that the local Boltzmann succession rule decreased uncertainty and unpredictability the fastest, both with respect to a stringency parameter (β) regarding adherence to cross-impact judgments and time. Arctan had the highest uncertainty and unpredictability and its improvements for uncertainty and unpredictability measures are very slow.

Fifth, we investigated the usefulness of the new information about transient system behavior afforded by stochastic CIB. To assess this, we compared our findings to a recent review of the demographic literature [33]. As part of his review, Jiang noted the agreement of the literature as well as the quality of the evidence (see [33] Fig 2). Consistent with the literature, all CIB succession rules identified a “demographic trap” as a stable attractor, where total fertility rate is high, growth of income per capita is low, and rates of educational attainment are low. Our model also found evidence that advancing educational attainment can pull a society out of the trap and toward a lower total fertility rate (consistent with Jiang’s assessment of high agreement and medium evidence in the literature). However, local arctan succession showed this effect to be less certain if economic growth does not occur as well. This finding jibes with the demographic literature and adds further contextualization. Jiang assessed the literature for the pairwise influence from economic growth to population growth to be unclear (or subject to a society’s stage of development and urbanization), with medium agreement and robust evidence. For the pairwise influence from economic growth to educational attainment, Jiang assessed the literature to show a dominantly positive relationship with medium agreement and medium evidence. Our CIB model with local arctan succession suggests that for high-fertility and low-income countries, decreases in fertility are more likely to succeed if efforts to enhance educational attainment occur while the economy grows as well.

We conclude that the visualization of complete system successions as Markov chains is a useful improvement for CIB analysis, as it provides three types of new information about scenarios: (1) a visual map of scenarios’ relationship to each other; (2) how easy (or difficult) it may be for the system to transition from any particular scenario to another; and (3) potential paths between scenarios, which are suggestive of strategies for policy intervention. Potentially, the most informative way to use CIB to retain the information about both system attractors and transition paths is to use the following succession rules in tandem: the adiabatic rule (deterministic), the local Boltzmann rule (stochastic), and the local arctan rule (stochastic).

In addition, stochastic succession opens new possibilities for continued research, and we see two major avenues. First, we found that basins of attraction are retained across succession rules with moderate or low entropy. However, when entropy is too high, some valuable information about attractors will be lost. Further investigations of entropy thresholds could be done by varying the stringency parameter, β. Second, whole lines of study could be devoted to how knowledge users, such as decision makers, respond to CIB Markov chains. Potentially, these visualizations make the abstractness of alternative possible outcomes for “the future” more concrete, as well as opportunities for interventions to take pathways that lead to desirable futures (or alternatively, to avoid undesirable futures). This final avenue could have substantial implications for improving decision making under uncertainty.

## Supporting information

S1 Fig Comparison of forecasts for the Boltzmann, arctan, and logistic local succession rules for the Population-Income-Education example as a function of stringency. Left: Comparison from the perspective of long-range uncertainty. Right: Comparison from the perspective of unpredictability of forecasts.
(TIF)

S2 Fig Comparison of finite-time forecasts for the Boltzmann, arctan and logistic local succession rules for the Population-Income-Education example. We start from a uniform distribution (maximum uncertainty) and evolve with. Left panel: Comparison from the perspective of scenario uncertainty. Right panel: Comparison from the perspective of unpredictability of successions.
(TIF)

S3 Fig Markov chain (with 2% filter) of a 6-descriptor example with the local Boltzmann succession rule (beta = 2). The 6-descriptor case is the Somewhereland example discussed in the manual for [27]. The color of the nodes represents economic conditions with green representing “shrinking”, red representing “stagnant” and blue representing “dynamic”. Economic conditions were selected for illustrative purposes only. Other descriptors could also be used as partition criteria to study their distribution in the network.
(TIF)

S1 File (DOCX)
