# Supplementary figures and images for: Pathways for socio-economic system transitions expressed as a Markov chain
Source: PLoS One. 2023 Jul 31;18(7):e0288928. doi: 10.1371/journal.pone.0288928 (PMC10389699; doi:10.1371/journal.pone.0288928)

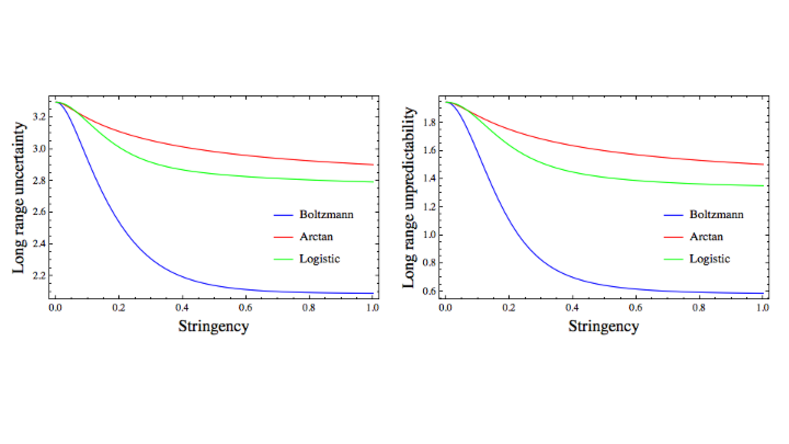

Supplement: S1 Fig — Left: Comparison from the perspective of long-range uncertainty. Right: Comparison from the perspective of unpredictability of forecasts. (TIF) [file pone.0288928.s001.tif]

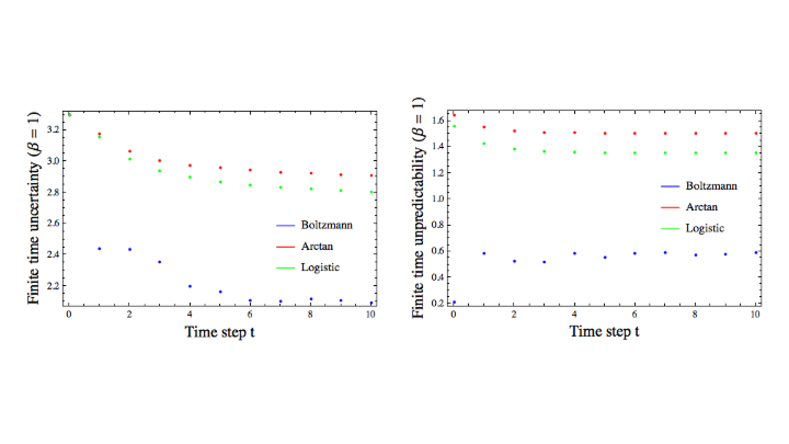

Supplement: S2 Fig — We start from a uniform distribution (maximum uncertainty) and evolve with. Left panel: Comparison from the perspective of scenario uncertainty. Right panel: Comparison from the perspective of unpredictability of successions. (TIF) [file pone.0288928.s002.tif]

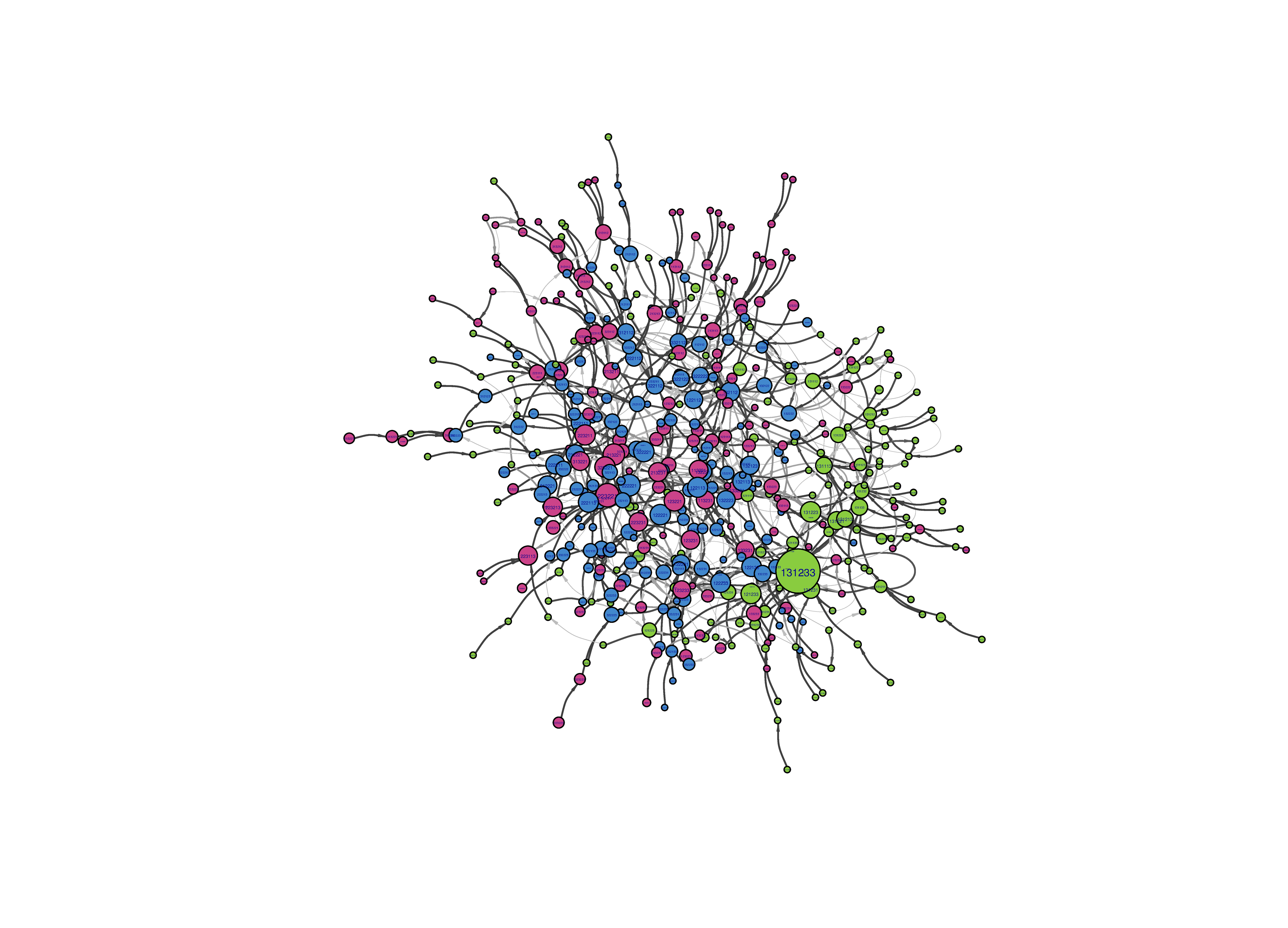

Supplement: S3 Fig — The 6-descriptor case is the Somewhereland example discussed in the manual for [27]. The color of the nodes represents economic conditions with green representing “shrinking”, red representing “stagnant” and blue representing “dynamic”. Economic conditions were selected for illustrative purposes only. Other descriptors could also be used as partition criteria to study their distribution in the network. (TIF) [file pone.0288928.s003.tif]
