# Supplementary material for: Pathways for socio-economic system transitions expressed as a Markov chain
Source: PLoS One. 2023 Jul 31;18(7):e0288928. doi: 10.1371/journal.pone.0288928 (PMC10389699; doi:10.1371/journal.pone.0288928)
Supplement: S1 File — (DOCX) [file pone.0288928.s004.docx]

# **Supporting information**

**Supporting Information Section 1: Data, Tools and Processing**

All techniques and methods discussed in the main article are implemented in the StochasticCIB R package, which can be found at <https://github.com/alastair-JL/StochasticCIB>

To install StochasticCIB, open your prefered R environment (for example Rstudio) and type:

install.packages("devtools")

library("devtools")

install_github('alastair-JL/StochasticCIB')

library(StochasticCIB)

The simple three-factor impact matrix discussed in this paper was constructed for [13] and first published in standardized form by [23]. The impacted matrix can be loaded into R:

data(ExampleCIBdata)

Alternatively, users may input their own impact matrix using:

CIBobject<- InputCibBanner()

Once a Cross Impact Balance matrix has been created, state adjacency and pairwise consistency scores are calculated using

TransScoresAdj<-MakeScoreMatrix(ExampleCIBdata)

Particular transition rules are implemented using the corresponding transition function, for example:

boltzTrans<-LocalBoltzmann(ExampleCIBdata)

TransScoresAdj<-MakeScoreMatrix(ExampleCIBdata)

TransMaxAdj<-TransToMaxAdj(ExampleCIBdata,TransScoresAdj)

Additional help can be found via:

help("StochasticCIB")

help(“TransitionCalculators”)

Creation of figures was completed via igraph [30].

**Supporting Information Section 2: Entropy production**

One aspect of our original investigation now suppressed in the main document is the study of entropy production: the rate at which uncertainty is generated by a system. Given a forecast $F$, how uncertain is the successor $z'$ of a given scenario $z$? This question can be addressed by computing the *entropy production* of the Markov chain:

$h_{\mu}[F] = -\sum_{z} F(z) \sum_{z^{'}} T(z\to z') \text{log} T(z\to z')$. (S.1)

Entropy production is calculated in three steps. First, consider each scenario $z$, weighted by its probability $F(z)$ in the forecast. Second, evaluate the level of randomness in successions away from $z$ (for example, coin flipping is completely unpredictable, while successions that are almost certain or almost impossible have a low level of randomness). Third, sum the contributions of all scenarios. The resulting number is the entropy production, and it is interpreted as the *mean unpredictability* of successions given the forecast $F$.

The unpredictability of a system can be used to distinguish between (for example) a cycle of three states each succeed by one another in a pre-determined sequence (highly predictable), and a wide basin containing three equally probably states, where at any given time step we might jump to any of the three (unpredictable). Systems with low long-term unpredictability tend to “settle”—if we wait long enough, we will be able to start making strong predictions. Conversely, systems with high long-term unpredictability will remain hard to predict indefinitely. With both uncertainty and unpredictability defined, we now move on to examine both for each of our three local stochastic rules. Here we explore the relationships between uncertainty and unpredictability, time, and our stringency parameter ($\beta$).

From Fig S1, we observe several things. First, both long-term uncertainty and long-term unpredictability decrease as the $\beta$ parameter increases. This makes sense, as a higher $\beta$ increases the differential between positive and negative impact gradients (c.f. Fig 3 in the main paper). Also, uncertainty and unpredictability both reach a plateau as $\beta$ goes to infinity; this means that even for “high stringency”, all three weight functions (Boltzmann, arctan and logistic) behave in a highly stochastic manner.

In Fig S2, we also consider the uncertainty of our predictions after N time steps—that is, after N time steps, how definite are our forecasts, and how unpredictable do we expect the system to be at that time? As time increases, we know that the Markov chain picks a subset of scenarios as more probable, hence uncertainty decreases over time. This is seen for all three weight functions (left panel). This notwithstanding, transitions remain stochastic at all times, and unpredictability remains approximately constant, as is apparent in the right panel.

**Fig S1. Comparison of forecasts for the Boltzmann, arctan, and logistic local succession rules for the Population-Income-Education example as a function of stringency.** Left: Comparison from the perspective of long-range uncertainty. Right: Comparison from the perspective of unpredictability of forecasts.

**Fig S2. Comparison of finite-time forecasts for the Boltzmann, arctan and logistic local succession rules for the Population-Income-Education example.** We start from a uniform distribution (maximum uncertainty) and evolve with $\beta=1$. Left panel: Comparison from the perspective of scenario uncertainty. Right panel: Comparison from the perspective of unpredictability of successions.

**Supporting Information Section 3: Detail on the Population-Income-Education descriptors**

As discussed in section 5.2 in the main paper, the mapping of our model results to a recent review of literature in human demography (Jiang 2014) should be done with care. This Appendix details a comparison of the concepts embedded in our CIB model (which are based on a subset of expert judgments collected by Schweizer and O’Neill 2014) to those discussed by Jiang (2014).

Schweizer and O’Neill (2014) contributed the results of an expert-based CIB model to the development of the Shared Socio-economic Pathways (SSPs). They explained that SSPs should be considered aggregated trends for each variable, averaged at the global level. In his review of demographic literature, Jiang (2014) explained that demographers have concluded that the interactions between the aforementioned descriptors differ across stages of economic development. For this reason, it’s important to consider *rates of change* to the variables rather than the levels of the variables themselves. Jiang explained that this is why he parsed the literature of bidirectional effects between Population, Income growth, and Educational attainment as being applicable to low-, medium-, and high-income countries.

Below we review the meanings of the states of each descriptor from Schweizer & O’Neill (2014) as well as the verbiage employed by Jiang (2014) in his review.

**Population:** Ranges for global population projections through 2100 were provided by Schweizer and O’Neill (2014); however, they also stated that alternative outcomes for average global fertility could be considered. Jiang (2014) reviewed the literature according to the latter concept, so we use that interpretation below.

- **High:** A high total fertility rate (TFR), leading to population growth over time. Looking across countries of the world (including those with high TFR at replacement due to local mortality, see Espenshade et al. 2003), a high TFR would be more than approximately 3 births per woman. For reference, The World Factbook (US CIA 2019) indicates that Niger has the highest TFR in the world at 6.49 births per woman.
- **Medium:** Between high and low TFR, i.e. 2.1 – 3 births per woman.
- **Low:** A low TFR, leading to population decline over time. The UN Population Division defines the TFR at replacement as approximately 2.1 births per woman. A low total fertility rate would be below this rate. Industrialized countries are already at this rate with many being below replacement.

**Income:** Ranges for global GDP per capita projections through 2100 were provided by Schweizer and O’Neill (2014) as well as annualized growth rates. Jiang (2014) cautions against interpreting the influence of income according to level; however, he parses his discussion of demographic literature by development stage. Below, we interpret the meaning of each state for income per capita by *levels that are proxies* for development stage. Benchmark statistics provided below are based on data from The World Bank.

- **High:** Fast economic growth, sustained at approximately 2.0% or more annually, resulting in GDP per capita levels equivalent to present-day high-income countries, or HIC (approximately $40,000 in constant 2010 USD). The key distinguishing characteristics for this state are that GDP per capita is sustained at approximately the level of HICs, and economic growth is sufficiently high to prevent mass unemployment.
- **Medium:** Slower economic growth, sustained at approximately 1.5% - 2.0% annually, resulting in GDP per capita levels *below* present-day HICs but improved over low-income countries. The key distinguishing characteristic is whether GDP per capita remains significantly below that of HICs. For reference, for the past 10 years, GDP per capita in China consistently grew at a very fast rate of more than 6%; however, in 2017, Chinese GDP per capita was approximately $7,000. The World Bank classifies China as an upper-middle-income country.
- **Low:** Economic stagnation or contraction, sustained at less than 1.5% annually, resulting in GDP per capita levels that are, at best, approximately half that of present-day HICs (approximately $20,000 in constant 2010 USD; for reference, the US Census Bureau (Fontenot et al. 2018) indicates that the poverty threshold for a single individual in the US under age 65 in 2017 was approximately $13,000). For reference, for the past 10 years, GDP per capita growth in low- and middle-income countries ranged from 1.1% - 5.9%; however, in 2017, GDP per capita in low- and middle-income countries was approximately $4,500. For the definition of this state, the key distinguishing characteristic is whether GDP per capita remains *less than half* that of HICs.

**Educational attainment:** Schweizer and O’Neill (2014) projected ranges for the share of the global population with post-primary schooling through 2100 based on data provided by IIASA. Jiang (2014) provides more context, explaining that access to mass education accelerates growth in the share of the population with improved educational attainment. Below, we interpret the meaning of each state with qualitative descriptions suggested by Jiang.

- **High:** Mass education is available at the primary and secondary school levels, with most youth participating (e.g. compulsory schooling in HIC)
- **Medium:** Mass education is available, but many youths do not access or complete it, particularly at the secondary school level
- **Low:** Mass education is *not* available, or is inaccessible, to many youths, resulting in literacy and educational completion rates that are below the global average. For reference, according to the UNESCO Institute for Statistics, in 2010, approximately 68% of the global population undertook lower secondary education.^[[1]](#footnote-1)^

**Supporting Information Section 4: Markov chain of six-descriptor system**

The main paper features a 3-descriptor system for illustrative purposes. The concept and tools developed in this project could also be applied to more complex systems with more descriptors and states. Below in Fig S3 is a visualization of a Markov chain applying the local Boltzmann succession rule to a 6-descriptor example (each descriptor having 2-3 states, which yields 486 possible scenarios).

**Fig S3. Markov chain (with 2% filter) of a 6-descriptor example with the local Boltzmann succession rule (beta = 2).** The 6-descriptor case is the Somewhereland example discussed in the manual for [27]. The color of the nodes represents economic conditions with green representing “shrinking”, red representing “stagnant” and blue representing “dynamic”. Economic conditions were selected for illustrative purposes only. Other descriptors could also be used as partition criteria to study their distribution in the network.

**References for Supporting Information**

Espenshade, T.J., Guzman, J.C., Westoff, C.F., 2003. The Surprising Global Variation in Replacement Fertility. Population Research and Policy Review 22, 575–583. <https://doi.org/10.1023/B:POPU.0000020882.29684.8e>

Fontenot, K., Semega, J., Kollar, M., 2018. Income and Poverty in the United States: 2017 (Current Population Reports No. P60-263). US Census Bureau, Washington, DC.

Jiang, L., 2014. Internal consistency of demographic assumptions in the shared socioeconomic pathways. Popul Environ 35, 261–285. <https://doi.org/10.1007/s11111-014-0206-3>

Schweizer, V.J., O’Neill, B.C., 2014. Systematic construction of global socioeconomic pathways using internally consistent element combinations. Climatic Change 122, 431–445. https://doi.org/10.1007/s10584-013-0908-z

The World Bank, 2019. DataBank: World Development Indicators [WWW Document]. URL https://databank.worldbank.org/data/indicator/NY.GDP.PCAP.CD/1ff4a498/Popular-Indicators# (accessed 5.27.19).

UNESCO Institute of Statistics, 2019. Data for the Sustainable Development Goals [WWW Document]. URL http://uis.unesco.org/ (accessed 6.3.19).

US Central Intelligence Agency, 2019. Country Comparison: Total fertility rate [WWW Document]. The World Factbook. URL https://www.cia.gov/library/publications/the-world-factbook/fields/356rank.html (accessed 6.3.19).

1. The global average stated here is based on data of poor quality and may be an overestimate. In 2010, data coverage across countries is only 33% (with this being a high response rate compared to other years of available data). Across countries, educational attainment at the level of lower secondary schooling ranged from approximately 12% for Guinea to over 90% for countries such as Australia, Russia, the US, and Switzerland. [↑](#footnote-ref-1)
